# Duplication of Symbiotic Lysin Motif Receptors Predates the Evolution of Nitrogen-Fixing Nodule Symbiosis^1^

**DOI:** 10.1104/pp.19.01420

**Published:** 2020-07-15

**Authors:** Luuk Rutten, Kana Miyata, Yuda Purwana Roswanjaya, Rik Huisman, Fengjiao Bu, Marijke Hartog, Sidney Linders, Robin van Velzen, Arjan van Zeijl, Ton Bisseling, Wouter Kohlen, Rene Geurts

**Affiliations:** a Laboratory of Molecular Biology, Department of Plant Sciences, Wageningen University, 6708 PB Wageningen, The Netherlands; b Centre of Technology for Agricultural Production, Agency for the Assessment and Application of Technology, 10340 Jakarta, Indonesia; c Biosystematics Group, Department of Plant Sciences, Wageningen University, 6708 PB Wageningen, The Netherlands

## Abstract

Rhizobium nitrogen-fixing nodule symbiosis occurs in two taxonomic lineages: legumes (Fabaceae) and the genus *Parasponia* (Cannabaceae). Both symbioses are initiated upon the perception of rhizobium-secreted lipochitooligosaccharides (LCOs), called Nod factors. Studies in the model legumes *Lotus japonicus* and *Medicago truncatula* showed that rhizobium LCOs are perceived by a heteromeric receptor complex of distinct Lys motif (LysM)-type transmembrane receptors named NOD FACTOR RECEPTOR1 (LjNFR1) and LjNFR5 (*L. japonicus*) and LYSM DOMAIN CONTAINING RECEPTOR KINASE3 (MtLYK3)-NOD FACTOR PERCEPTION (MtNFP; *M. truncatula*). Recent phylogenomic comparative analyses indicated that the nodulation traits of legumes, *Parasponia* spp., as well as so-called actinorhizal plants that establish a symbiosis with diazotrophic *Frankia* spp. bacteria share an evolutionary origin about 110 million years ago. However, the evolutionary trajectory of LysM-type LCO receptors remains elusive. By conducting phylogenetic analysis, transcomplementation studies, and CRISPR-Cas9 mutagenesis in *Parasponia andersonii*, we obtained insight into the origin of LCO receptors essential for nodulation. We identified four LysM-type receptors controlling nodulation in *P. andersonii*: *PanLYK1*, *PanLYK3*, *PanNFP1*, and *PanNFP2*. These genes evolved from ancient duplication events predating and coinciding with the origin of nodulation. Phylogenetic and functional analyses associated the occurrence of a functional NFP2-orthologous receptor to LCO-driven nodulation. Legumes and *Parasponia* spp. use orthologous LysM-type receptors to perceive rhizobium LCOs, suggesting a shared evolutionary origin of LCO-driven nodulation. Furthermore, we found that both PanLYK1 and PanLYK3 are essential for intracellular arbuscule formation of mutualistic endomycorrhizal fungi. PanLYK3 also acts as a chitin oligomer receptor essential for innate immune signaling, demonstrating functional analogy to CHITIN ELECITOR RECEPTOR KINASE-type receptors.

## Abstract (precis)

Four lysin motif receptor kinases controlling rhizobium nodule formation in the nonlegume Parasponia evolved after two ancient duplications.

Nitrogen availability is a critical factor for plant growth, but fixed nitrogen in the form of nitrate or ammonia in soils is limited. Plants have acquired different strategies to overcome this limitation. One such strategy is establishing a nodule endosymbiosis with nitrogen-fixing *Frankia* or rhizobium bacteria. Inside nodules, physiological conditions are created that allow the bacteria to convert atmospheric dinitrogen (N_2_) into ammonia that can be used by the plant. Carbohydrates of plant origin fuel this energy-demanding process. The unique character of nitrogen-fixing nodule symbiosis has raised the interest of plant researchers for more than a century, ultimately aiming to transfer this trait to nonleguminous crop species (Burrill and Hansen, 1917; Rogers and Oldroyd, 2014; Huisman and Geurts, 2019).

The *Frankia* spp. and rhizobium nitrogen-fixing nodulation trait occurs in 10 paraphyletic lineages within the orders Fabales, Fagales, Cucurbitales, and Rosales, collectively known as the nitrogen-fixing clade (Soltis et al., 1995). Based on phylogenomic comparisons of nodulating and nonnodulating plant species, it is hypothesized that the nitrogen-fixing nodule symbiosis with rhizobium or *Frankia* spp. bacteria has a shared evolutionary origin, dating to about 110 million years ago (Griesmann et al., 2018; van Velzen et al., 2018, 2019). Subsequently, the nodulation trait most probably was lost multiple times, which is associated with the pseudogenization of two key genes essential for nodule organogenesis and bacterial infection: the transcription factor *NODULE INCEPTION* (*NIN*) and the coiled-coil protein-encoding gene *RHIZOBIUM POLAR GROWTH* (Griesmann et al., 2018; van Velzen et al., 2018). These two genes likely experienced genetic adaptations, allowing them to function exclusively in nodulation. However, insight into the evolutionary trajectory of signaling receptors involved in the recognition of bacterial signals and subsequent activation of the pathways leading to nodule organogenesis and bacterial infection remains elusive.

The nitrogen-fixing nodulation trait is best studied in the legume models *Lotus japonicus* and *Medicago truncatula* (Fabaceae, Fabales). Both these legumes recognize their rhizobium microsymbionts by the structural characteristics of secreted lipochitooligosaccharides (LCOs; also known as Nod factors). Perception of these molecules triggers nodule development (Wang et al., 2012). LCO signaling is also the basis of rhizobium-induced nodulation in the nonlegume genus *Parasponia* (Cannabaceae, Rosales; Marvel et al., 1987; Op den Camp et al., 2011; van Velzen et al., 2018). Additionally, it was found that diazotrophic *Frankia* spp. strains of a basal taxonomic lineage (so-called cluster II strains) possess LCO biosynthesis genes, but the nodulating strains of two other taxonomic clusters do not (Pawlowski and Demchenko, 2012; Persson et al., 2015; Nguyen et al., 2016, 2019). LCOs, as well as chitin oligomers (COs), are also used by arbuscular mycorrhiza (AM) fungi to signal their hosts (Maillet et al., 2011; Genre et al., 2013). Perception of these AM signals requires a plant Lys motif (LysM)-type receptor that also is essential for chitin innate immune signaling, such as the CHITIN ELECITOR RECEPTOR KINASE1 (OsCERK1) in rice (*Oryza sativa*; Miyata et al., 2014; Zhang et al., 2015; He et al., 2019). This suggests that nodulating bacteria coopted LCO signaling from the widespread AM symbiosis and/or innate immune signaling (Parniske, 2008; Gough and Cullimore, 2011; Geurts et al., 2012).

Genetic and biochemical studies in *L. japonicus* and *M. truncatula* demonstrated that rhizobium LCOs are perceived specifically by a heteromeric complex containing two distinct LysM-type receptors, named NOD FACTOR RECEPTOR1 (LjNFR1) and LjNFR5 in *L. japonicus* and LYSM DOMAIN CONTAINING RECEPTOR KINASE3 (MtLYK3) and NOD FACTOR PERCEPTION (MtNFP) in *M. truncatula* (Limpens et al., 2003; Madsen et al., 2003; Radutoiu et al., 2003, 2007; Arrighi et al., 2006; Broghammer et al., 2012). Other receptors may modulate the LCO response, such as LjNFRe, a homolog of LjNFR1 in *L. japonicus* (Murakami et al., 2018). The LysM-type receptor family can be divided into two subclasses, named LYK and LYR, characterized by having a functional or dead kinase domain (Arrighi et al., 2006). Together, these make up 11 orthogroups, two of which include legume LCO receptors (Buendia et al., 2018). Within legumes, the orthogroup that includes LjNFR1/MtLYK3 (named LYK-I clade) expanded upon gene duplications, allowing functional separation of rhizobium-induced signaling, AM symbiosis, and chitin-triggered innate immune responses (De Mita et al., 2014; Bozsoki et al., 2017; Buendia et al., 2018; Gibelin-Viala et al., 2019). Likewise, *LjNFR5*/*MtNFP* (orthogroup LYR-IA) experienced a gene duplication early in the legume clade (Young et al., 2011; Buendia et al., 2018).

Data on symbiotic LysM-type receptors in nodulating nonlegumes are scarce. Only in *Parasponia andersonii* has a receptor functioning in nodulation been identified; named *PanNFP1*, it is a close homolog of *LjNFR5/MtNFP* (Op den Camp et al., 2011). Besides *PanNFP1*, *Parasponia* spp. possess a homologous receptor, named *NFP2*, that is more closely related to *LjNFR5/MtNFP* and transcriptionally activated in root nodules. Interestingly, this receptor is pseudogenized in nonnodulating Rosales species (van Velzen et al., 2018). To obtain insight into the evolution of LysM-type LCO receptors that are essential for nodulation, we used *P. andersonii* as a comparative system to legumes. The genus *Parasponia* represents five tropical tree species, which form nitrogen-fixing nodules with LCO-producing rhizobium species that also nodulate legumes (van Velzen et al., 2018). *Parasponia* spp. and legumes diverged at the root of the nitrogen-fixing clade more than 100 million years ago (Li et al., 2015; van Velzen et al., 2019). The microbial symbionts of the ancestral nodulating plants remain elusive, and it is probable that *Parasponia* spp. and legumes accepted rhizobium as a microbial partner in parallel (van Velzen et al., 2019). In line with this, the genus *Parasponia* provides a unique comparative system to obtain insight into the evolutionary trajectories of different LCO receptors that are essential for nodulation.

## RESULTS

### Phylogeny Reconstruction of Orthogroups Representing LysM-Type LCO Receptors

To obtain insight into the LysM-type receptor family of *P. andersonii*, we analyzed it phylogenetically. We identified 16 *P. andersonii* genes encoding putative LysM-type receptors that grouped in all except one known orthogroups (Supplemental Fig. S1; Supplemental Table S1). Genetic studies in legumes uncovered only two orthogroups that contain proteins with a known function in rhizobium LCO signaling; these are named LYK-I and LYR-IA (Buendia et al., 2018). *P. andersonii* has two gene copies in both these orthogroups.

LYK-I is the largest orthogroup, containing the functional legume LCO receptors *MtLYK3/LjNFR1* and *LjNFR*e (Limpens et al., 2003; Radutoiu et al., 2003; Murakami et al., 2018). Besides these, the LYK-I orthogroup also includes chitin innate immune receptors of *M. truncatula MtLYK9/MtCERK1*, *L. japonicus* *LjCERK6*, Arabidopsis (*Arabidopsis thaliana*) *AtCERK1*, tomato (*Solanum lycopersicum*) *SlLYK1*, and rice *OsCERK1* (Limpens et al., 2003; Miya et al., 2007; Wan et al., 2008; Shimizu et al., 2010; Miyata et al., 2014; Zhang et al., 2015; Bozsoki et al., 2017; Carotenuto et al., 2017; Liao et al., 2018; Gibelin-Viala et al., 2019; He et al., 2019). OsCERK1 and MtLYK9/MtCERK1 have also been found to function in AM symbiosis (Miyata et al., 2014; Zhang et al., 2015; Feng et al., 2019; Gibelin-Viala et al., 2019). Two *P. andersonii* genes are part of this orthogroup, named *PanLYK1* and *PanLYK3*.

**Figure 1.**
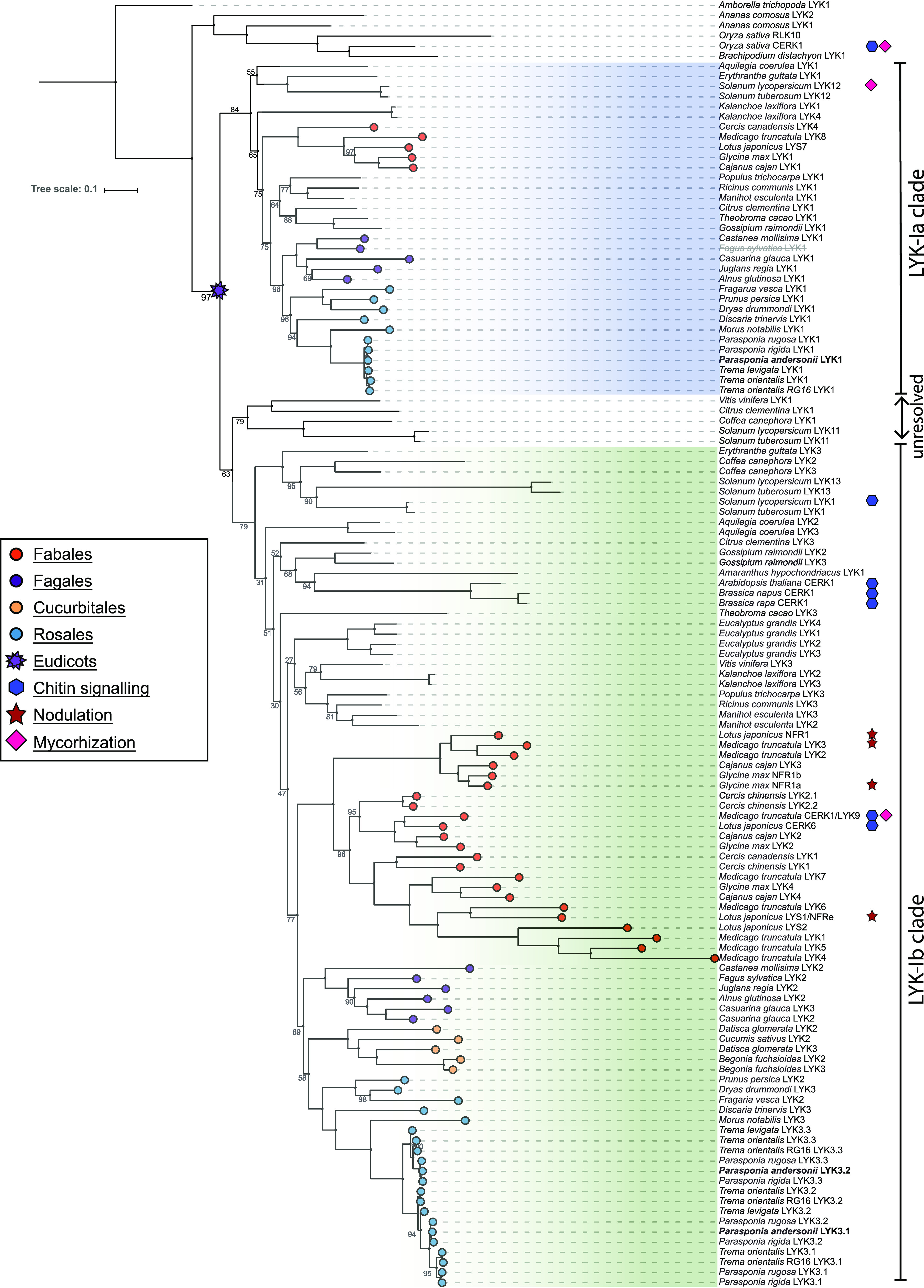
Phylogeny reconstruction of the LYK-I orthogroup, containing known CO and LCO receptors, based on 127 sequences from 47 species. Two main subgroups are recognized in eudicots, LYK-Ia (blue) and LYK-Ib (green). Note the presence of both variants in *Aquilegia coeralia*, a basal eudicot in the Ranunculales. A subset of proteins is unresolved. *P. andersonii* proteins are in boldface. Genuses *Parasponia* and *Trema* LYK3.1 and LYK3.2 represent protein variants of LYK3. Deduced pseudoproteins are depicted in gray/strikethrough. Proteins with known functions in nodulation, mycorrhization, and/or chitin innate immune signaling are indicated. Bootstrap values indicate IQ-tree UF-bootstrap support%; values >98 are not shown. The scale bar represents substitutions per site. A complete list of species and accession numbers can be found in Supplemental Data Set S1.

**Figure 2.**
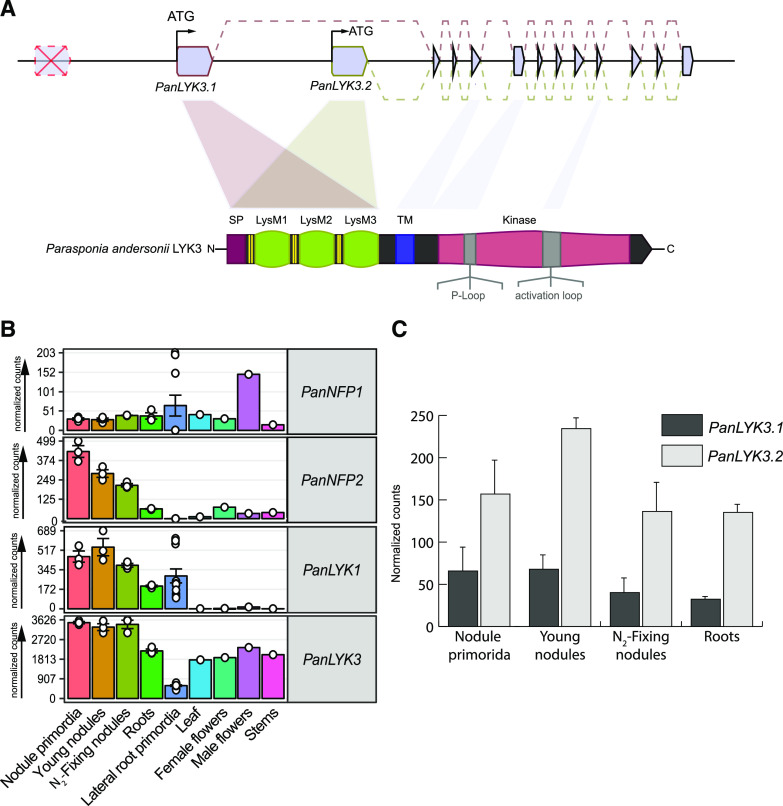
Gene structure and expression of *P. andersonii PanLYK3*. A, Structure of the *PanLYK3* gene model and encoded proteins. *PanLYK3* possesses two transcriptional start sites resulting in two protein variants, which differ in the extracellular region containing the LysM domains and are encoded by exon 1. The red cross indicates a third upstream copy of exon 1 lost in *P. andersonii* but maintained in other *Parasponia* and *Trema* species. B, Expression profile of *PanNFP1*, *PanNFP2*, *PanLYK1*, and *PanLYK3* in different plant tissues. Expression is given in DESeq2-normalized read counts; error bars represent se of biological replicates. Circles represent individual expression levels. The analysis is based on data presented by van Velzen et al. (2018). C, Relative expression of the *PanLYK3.1* and *PanLYK3.2* transcriptional variants based on RNA sequencing reads splicing into the second exon. Data are represented as means ± se (*n* = 3). The analysis is based on data presented by van Velzen et al. (2018).

A more exhaustive phylogenetic reconstruction was conducted using gene orthologs of additional species to obtain insight into the evolutionary relationships of these genes when compared with LCO and CO receptors. Notably, LysM-type receptors of the recently sequenced nodulating actinorhizal plants and nonnodulating relatives were included (Griesmann et al., 2018). The resulting phylogeny largely resembled rosid species trees as reconstructed on the basis of plastid-coding genes (Wang et al., 2009; Gonçalves et al., 2019). Our analysis revealed that *PanLYK1* and *PanLYK3* originated from an ancient duplication, dividing this orthogroup into two subgroups that we named LYK-Ia and LYK-Ib. This duplication does not coincide with the birth of the nitrogen-fixing clade but rather occurred in an ancestral eudicot (Fig. 1; Supplemental Data Set S1). The only studied member in the LYK-Ia orthogroup is tomato *SlLYK12*, and knockdown of this gene by virus-induced gene silencing substantially reduces mycorrhizal colonization (Liao et al., 2018). The LYK-Ib clade represents several functionally characterized genes, including the chitin innate immune receptors and legume rhizobium LCO receptors. Legumes exhibit an increased number of genes in the LYK-Ib subclade, which are the result of tandem duplications (Limpens et al., 2003; Radutoiu et al., 2003; Zhu et al., 2006). These duplications may have driven neofunctionalization of LCO receptors in legumes (De Mita et al., 2014). In the genus *Parasponia*, no gene duplications occurred in the LYK-Ib clade (represented by *PanLYK3*) or in the LYK-Ia clade (represented by *PanLYK1*). In contrast, *P. andersonii PanLYK3* experienced a duplication of exclusively the first exon. To determine whether this duplication is specific for the *Parasponia* genus, we analyzed the *LYK3* genomic region of two additional *Parasponia* spp. and three nonnodulating species of the closely related genus *Trema*. This revealed that the duplication of *LYK3* exon 1 is present in all species investigated and occurred twice, where the most distal exon 1 copy was lost in *P. andersonii* (Fig. 2A; Supplemental Fig. S2A). The encoded pre-mRNAs both splice into a shared second exon (Fig. 2). Each exon 1 copy contains a putative transcription and translation start site, which allows for differential expression of the variants (Fig. 2, B and C). Genes of the LYK-I clade have a highly conserved intron-exon structure (Zhang et al., 2009). In most cases, the first exon encodes the extracellular domain comprising the signal peptide and three LysM domains. Therefore, the *P. andersonii PanLYK3* gene encodes two protein variants, named PanLYK3.1 and PanLYK3.2, that differ in their extracellular domain (Supplemental Fig. S2B).

**Figure 3.**
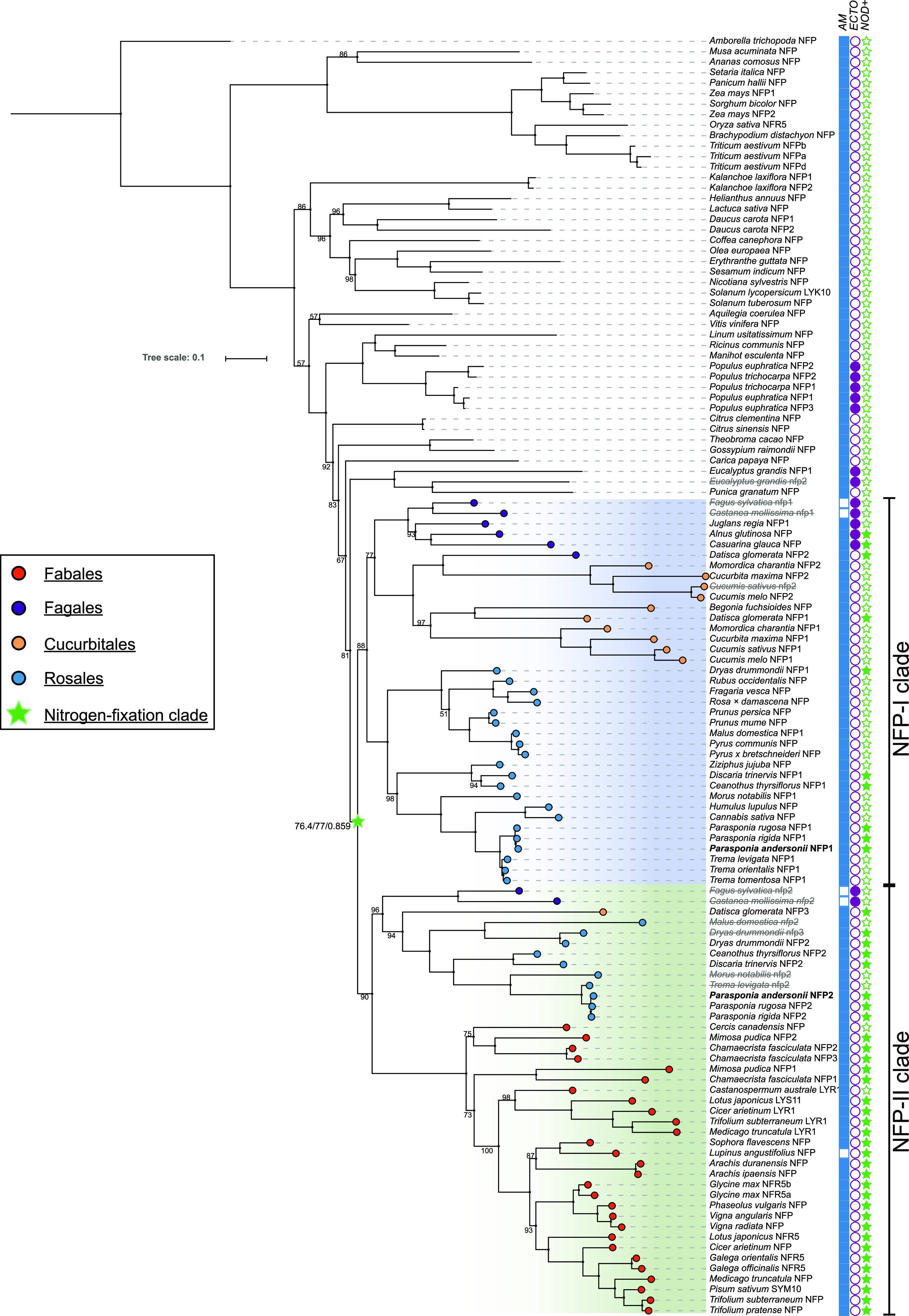
Phylogeny reconstruction of the LYR-IA orthogroup, containing known legume LCO receptors, based on 122 sequences from 87 species. A gene duplication in the root of the nitrogen-fixing clade is recognized, resulting in two subclades named NFP-I (blue) and NFP-II (green). The symbiotic capacities of the species are marked by filled (positive) and unfilled (negative) symbols: AM symbiosis (blue squares), ectomycorrhizal symbiosis (purple circles), and nodulation (green stars). *P. andersonii*PanNFP1 and PanNFP2 are in boldface. Deduced pseudoproteins are depicted in gray/strikethrough. Values indicate IQ-tree UF-bootstrap support%; values >98 are not shown. Branch support for the nitrogen-fixing clade indicates aSH-aLRT/UF-Bootstrap/approximate Mr.Bayes support, respectively. The scale bar represents substitutions per site. A list of species and accession numbers can be found in Supplemental Data Set S2.

The LYR-IA orthogroup represents the legume LCO receptors MtNFP, LjNFR5, and pea (*Pisum sativum*) PsSYM10 (Madsen et al., 2003; Arrighi et al., 2006; Buendia et al., 2016; Miyata et al., 2016). Previously, we have shown that *Parasponia* spp. harbor two genes in this orthogroup, *PanNFP1* and *PanNFP2* in *P. andersonii*, of which the latter is more closely related to *MtNFP*/*LjNFR5* (van Velzen et al., 2018). *PanNFP1* and *PanNFP2* originated from an ancient duplication. Phylogenetic reconstruction, including additional nodulating and nonnodulating species, supported the occurrence of NFP-I and NFP-II subclades in the LYR-IA orthogroup and showed that this duplication associates with the origin of the nitrogen-fixing clade (Fig. 3; Supplemental Data Set S2). Several actinorhizal species possess gene copies in both NFP subclades, including *Datisca glomerata*, *Dryas drummondii*, and *Ceanothus thyrsiflorius*. All these species nodulate with diazotrophic *Frankia* spp. of taxonomic cluster II, which possess LCO biosynthesis genes. An NFP-II-type orthologous gene is notably absent in actinorhizal species that are exclusively nodulated by *Frankia* spp. of cluster I or cluster III that lack LCO biosynthesis genes, such as *Alnus glutinosa* and *Casuarina glauca* (Fig. 3; Pawlowski and Demchenko, 2012; Griesmann et al., 2018; Salgado et al., 2018; Nguyen et al., 2019). In line with what was reported for the nonnodulating Rosales species (van Velzen et al., 2018), NFP-II-type pseudogenes can be found in the genomes of the nonnodulating Fagales spp. Chinese chestnut (*Castanea mollissima*) and European beech (*Fagus sylvatica*). This shows a strict association of the presence of a functional NFP-II-type gene and LCO-driven nodulation, suggesting that the NFP-II subclade represents LCO receptors that function exclusively in nodulation.

### *P. andersonii* PanNFP1, PanNFP2, PanLYK1, and PanLYK3 Can Perceive Rhizobium LCOs

Based on the orthologous relation to legume LCO receptors, we considered *PanLYK3* (both variants) and *PanNFP2* as the most likely candidates to encode rhizobium LCO receptors in *P. andersonii*. We noted that, in contrast to *PanLYK3*, *PanLYK1* is exclusively expressed in roots and nodule tissue (Fig. 2B), suggesting that this gene may also function in a symbiotic context. Therefore, we decided to include this gene in further studies. Finally, we also included *PanNFP1*, since an earlier study based on RNA interference (RNAi) in transformed *P. andersonii* roots showed that this gene functions in nodulation (Op den Camp et al., 2011). To test whether these four *P. andersonii* genes can function as rhizobium LCO receptors, we conducted two complementary experiments. First, we introduced *P. andersonii* receptor pairs into a *L. japonicus* *Ljnfr1;Ljnfr5* double mutant, aiming to determine whether these *P. andersonii* receptors can transcomplement for LCO-induced Ca^2+^ oscillation. Second, we generated CRISPR-Cas9 knockout mutants in *P. andersonii* to study their role in nodulation.

**Figure 4.**
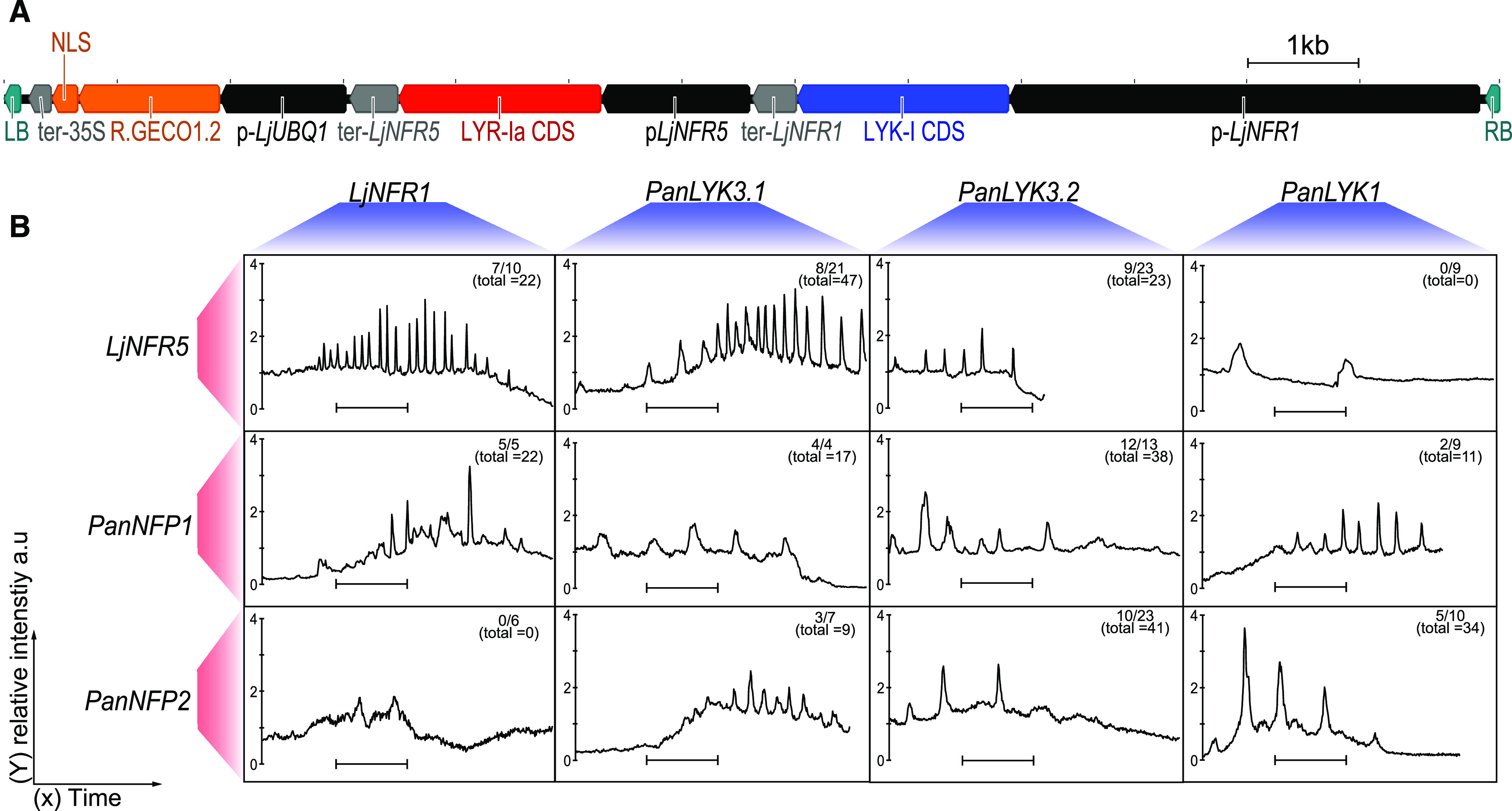
*P. andersonii PanNFP1*, *PanNFP2*, *PanLYK1*, and *PanLYK3* complement a *L. japonicus* *Ljnfr1;Ljnfr5* mutant for rhizobium-induced Ca^2+^ oscillation. A, Schematic representation of the T-DNA region of the binary construct used for *A. rhizogenes*-based root transformation of a *L. japonicus* *Ljnfr1;Ljnfr5* double mutant. cDNA clones of LYK-I (marked blue) or LYR-Ia (marked red) type genes were cloned in identical fashion. cDNA clones were inserted between native promoter (marked black) pLjNFR1 (4,171 bp) or pLjNFR5 (1,314 bp) and native terminator (marked gray) sequences ter-LjNFR1 (394 bp) or ter-NFR5 (432 bp). pLjUBQ1::R.GECO1.2-nls:CaMV35S-ter (marked orange) was used to visualize nuclear calcium oscillation. The left border (LB) and right border (RB; marked green) flank the T-DNA region. B, Representative traces of nuclear Ca^2+^ oscillation, as observed in different combinations of LYK-I (red) and LYR-Ia (blue) type receptors introduced in a *L. japonicus* *Ljnfr1;Ljnfr5* double mutant. Note that the receptor combinations *PanLYK1;LjNFR5* and *LjNFR1;PanNFP2* did not complement for Ca^2+^ oscillation. Traces were recorded ∼10 min postapplication of LCOs extracted from *M. loti* R7A (∼10^−9^ m). Numbers denote spiking roots versus the number of roots analyzed. The numbers in parentheses denote the total numbers of spiking nuclei observed. The *y* axis is the relative fluorescence intensity compared with the defined baseline in arbitrary units (a.u.). The scale bar = 10 min.

We selected *L. japonicus* for transcomplementation studies because its microbial host *Mesorhizobium loti* strain R7A can also nodulate *P. andersonii* (Supplemental Fig. S3, A–C). By using *Agrobacterium rhizogenes*-mediated root transformation, we tested six combinations of *P. andersonii* heterodimeric receptor pairs under the control of the promoter and terminator of LjNFR1 and LjNFR5 (Fig. 4). These promoters were shown to be functional in complementation of the *L. japonicus* *Ljnfr1-1;Ljnfr5-2* double mutant (Supplemental Fig. S3, D–H). For the transcomplementation constructs, we included the nucleus-localized calcium sensor R-GECO1.2, allowing visualization of nuclear Ca^2+^ oscillations (Zhao et al., 2011). In wild-type *L. japonicus* roots, Ca^2+^ oscillation was most strong in young root hair cells, whereas this response is not recorded in the *Ljnfr1-1;Ljnfr5-2* double mutant (Supplemental Fig. S3, I and J; Supplemental Movie S1; Miwa et al., 2006). Analyzing the transgenic roots expressing *P. andersonii* receptor combinations revealed that nine out of 11 tested combinations elicit Ca^2+^ oscillation, although less regular in shape and frequency when compared with the positive control (Fig. 4B; Supplemental Movie S2). Interestingly, the receptor combinations *PanLYK1;LjNFR5* and *LjNFR1;PanNFP2* did not elicit any Ca^2+^ oscillation response, whereas both *P. andersonii* receptors are, at least partially, functional as an *M. loti* LCO receptor when combined with a *P. andersonii* counterpart (Fig. 4B). Upon inoculation with *M. loti* R7A, only nodule-like structures were observed on roots transcomplemented with different *P. andersonii* receptor combinations (4 weeks postinoculation [wpi]) but not with heterologous receptor pairs (Supplemental Table S2). We sectioned the largest nodule-like structures, which were present on *PanLYK3.2;PanNFP2* and *PanLYK1;PanNFP1* transformed plants. This showed the absence of intracellular rhizobium infections (Supplemental Fig. S3, K–P). Taken together, the transcomplementation studies of a *L. japonicus* *Ljnfr1;Ljnfr5* mutant indicated that all four *P. andersonii* receptors, *PanLYK1*, *PanLYK3*, *PanNFP1*, and *PanNFP2*, have the potential to function as receptors for *M. loti* LCOs, but none could fully transcomplement a *Ljnfr1-1;Ljnfr5-2* double mutant for nodulation.

### *P. andersonii* PanNFP1, PanNFP2, PanLYK1, and PanLYK3 Function in Nodulation

We recently established an efficient *Agrobacterium tumefaciens*-mediated transformation protocol for *P. andersonii*, which allows the generation of CRISPR-Cas9 mutant plantlets in an ∼3-month time frame (van Zeijl et al., 2018; Wardhani et al., 2019). This enabled us to test by mutagenesis whether *PanLYK1*, *PanLYK3*, *PanNFP1*, and *PanNFP2* are essential for rhizobium-induced nodule formation. We aimed to generate small deletions of 100 to 300 bp in the area covering the LysM domains by using two or three single guide RNAs (sgRNAs) that have no potential high-identity off-targets. In the case of *PanLYK3*, the transmembrane domain was targeted in order to mutate both alternative start variants. Additionally, we targeted specifically *PanLYK3.1* and *PanLYK3.2* by designing specific guides on the first exon. Selected single guides only had off-targets with at least three mismatches or two insertions/deletions (indels), based on alignments to the *P. andersonii* reference genome. Shoots regenerated after *A. tumefaciens*-mediated cocultivation were genotyped using PCR and subsequent sequence analysis to detect potential mutations at the CRISPR target sites. Only T0 shoots with a >75-bp deletion between the two target sites or edits generating a frameshift were considered for propagation and subsequent further evaluation. At least two independent mutant alleles were generated per gene, with the exception of *PanLYK3.1*, for which only a single suitable allele could be identified (Supplemental Data Set S3). Putative off-target sites that occur in coding sequence regions were amplified by PCR and subsequently sequenced by Sanger sequencing. Subsequently, *PanNFP1* was sequenced in *PanNFP2* lines and *PanNFP2* was sequenced in *PanNFP1* lines (Supplemental Data Set S3). No off-target mutations at these locations were identified. The selected tissue culture lines were in vitro propagated and rooted, so they could be used for experimentation.

**Figure 5.**
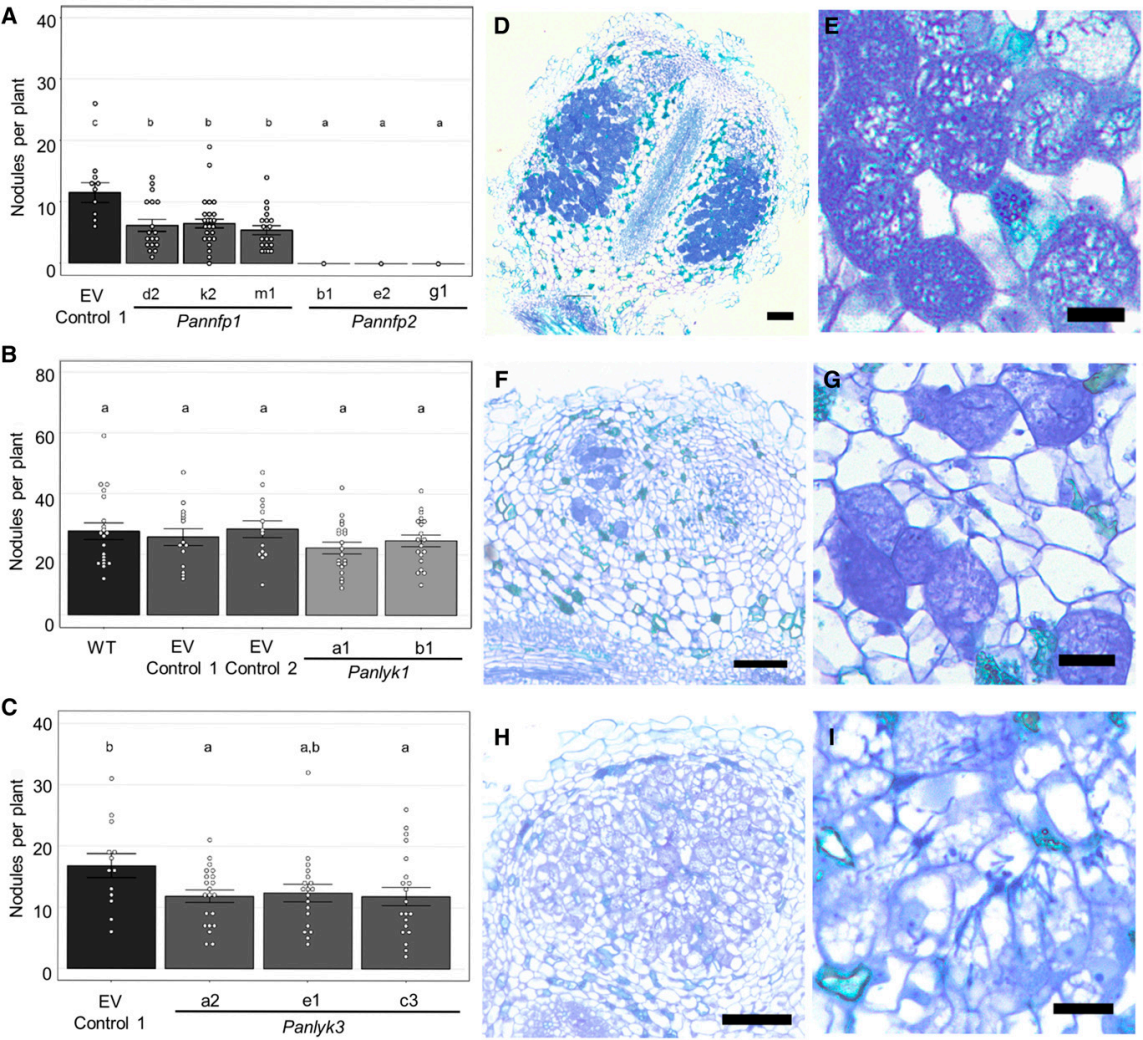
*P. andersonii Pannfp1*, *Pannfp2*, and *Panlyk3* mutants are affected in nodulation. Data are represented as means ± se, and circles represent individual data points. Lowercase letters denote statistical significance based on one-way ANOVA and Tukey’s posthoc contrast (*P* > 0.05). A, Nodule numbers in *P. andersonii* CRISPR-Cas9 mutant lines *Pannfp1* d2 (*n* = 18), k2 (*n* = 31), and m1 (*n* = 19) and *Pannfp2* b1 (*n* = 19), e2 (*n* = 10), and g1 (*n* = 9) at 5 wpi with *M. plurifarium* BOR2. EV Control 1 (*n* = 12) represents a positive control line transformed with a binary vector not containing sgRNAs. B, Nodule numbers in *P. andersonii* CRISPR-Cas9 mutant lines *Panlyk1* a1 (*n* = 19) and b1 (*n* = 20) at 5 wpi with *M. plurifarium* BOR2. EV Control 1 (*n* = 14) and EV Control 2 (*n* = 14) represent two independent positive control lines transformed with a binary vector not containing sgRNAs. WT (*n* = 20) represents untransformed plantlets. C, Nodule numbers in *P. andersonii* CRISPR-Cas9 mutant lines *Panlyk3* a2 (*n* = 21), c3 (*n* = 21), and e1 (*n* = 19) at 5 wpi with *M. plurifarium* BOR2. EV Control 1 (*n* = 14). EV Control 1 represents a positive control line transformed with a binary vector not containing sgRNAs. D to I, Toluidine Blue-stained sections of representative nodules grown with *M. plurifarium* BOR2. D, Wild-type *P. andersonii* transformed with an EV-1 construct expressing Cas9. E, Infected nodule cells containing fixation threads formed on EV-1 plants. F, Infected nodule of *Panlyk3* line e2. Note patches of infected cells. G, Infected nodule cells of *Panlyk3* line e2 containing fixation threads. H, Empty nodule of *Panlyk3* line e2. Note the absence of fully infected cells. I, Nodule cells of *Panlyk3* line e2 containing infection threads but no fixation threads. Bars = 100 μm (D, F, and H) and 20 μm (E, G, and I).

We compared the nodulation phenotypes of *Panlyk1*, *Panlyk3*, *Pannfp1*, and *Pannfp2* knockout mutants in independent experiments using empty vector (EV) transformed lines as controls (Fig. 5; Supplemental Fig. S4). All three independent *Pannfp2* mutant lines showed to be unable to form nodules or nodule-like structures (5 wpi) with strain *Mesorhizobium plurifarium* BOR2, demonstrating the requirement for this gene in the nodulation trait (Fig. 5A). Additionally, we noted a reduced nodulation efficiency of all three independent *Pannfp1* mutant lines. This is in line with earlier findings using RNAi to target *PanNFP1* in *A. rhizogenes*-transformed *P. andersonii* roots (Op den Camp et al., 2011), demonstrating that *Pannfp1* controls nodulation efficiency but is not essential for rhizobium intracellular infection. Previously, we reported that *PanNFP1* RNAi nodules have a strong infection phenotype when inoculated with the *Sinorhizobium fredii* strain NGR234 (Op den Camp et al., 2011). We did not observe such an infection phenotype in nodules induced by *M. plurifarium* BOR2 on *Pannfp1* knockout mutant plants (Supplemental Fig. S4). In order to determine whether the *Pannfp1* infection phenotype is strain dependent, we nodulated plants also with *S. fredii* NGR234. This strain was shown to be less optimal under the chosen conditions (Agroperlite supplemented with EKM medium [see “Materials and Methods”)] and *S. fredii* NGR234.pHC60 at OD = 0.05). In an effort to optimize nodulation efficiency with this strain, we used river sand and scored nodulation 8 wpi. Under these conditions, no difference between *Pannfp1* and the EV control was observed. Nodules formed on *Pannfp1* mutant plants were infected normally (Supplemental Fig. S4).

Similarly to *Pannfp1* mutant plants inoculated with *M. plurifarium* BOR2, we found a reduced nodulation efficiency in *P. andersonii Panlyk3* knockout mutants but not in *Panlyk3.1* and *Panlyk3.2* variant-specific mutant alleles, nor in *Panlyk1* mutants (Fig. 5; Supplemental Fig. S4). To determine whether nodules formed on *Panlyk1* and *Panlyk3* mutants have an infection phenotype, we analyzed thin sections. In contrast to legumes, *P. andersonii* does not guide rhizobia in infection threads toward the nodule primordia. Instead, rhizobia enter via apoplastic cracks in epidermis and cortex and only form infection threads to penetrate nodule cells. Once inside, infection threads develop into fixation threads, which are wider, having two phyla of bacteria aligned compared with one in infection threads, and possess a thinner cell wall (Lancelle and Torrey, 1984, 1985). *Panlyk1* mutant nodules showed no defects in infection thread structure or the transition from infection threads to fixation threads. In the case of *Panlyk3*, nodules were relatively small and had diverse phenotypes. Out of 45 sectioned nodules of the line *Panlyk3-e2*, 22 were infected like the wild type, 15 contained only infection threads but no fixation threads, and eight showed an intermediate phenotype with few infected cells (Fig. 5, F–I; Supplemental Fig. S4). To confirm that the infection phenotype is a result of a full *Panlyk3* knockout mutation, we sectioned 28 nodules of the independent knockout line *Panlyk3*-c3. This revealed similar results: 11 nodules normally infected, 11 contained only infection threads, and six nodules with an intermediate phenotype. Next, we determined whether this infection phenotype is controlled specifically by either *PanLYK3.1* or *PanLYK3.2*, which was shown not to be the case (Supplemental Fig. S4). As ∼50% of the nodules formed on the *P. andersonii Panlyk3* mutant plants displayed a wild-type phenotype, this suggests redundancy in gene functioning. Interestingly, *S. fredii* NGR234 could not nodulate *Panlyk3* mutants, which suggests that this strain is fully dependent on PanLYK3-controlled signal transduction (Supplemental Fig. S4).

**Figure 6.**
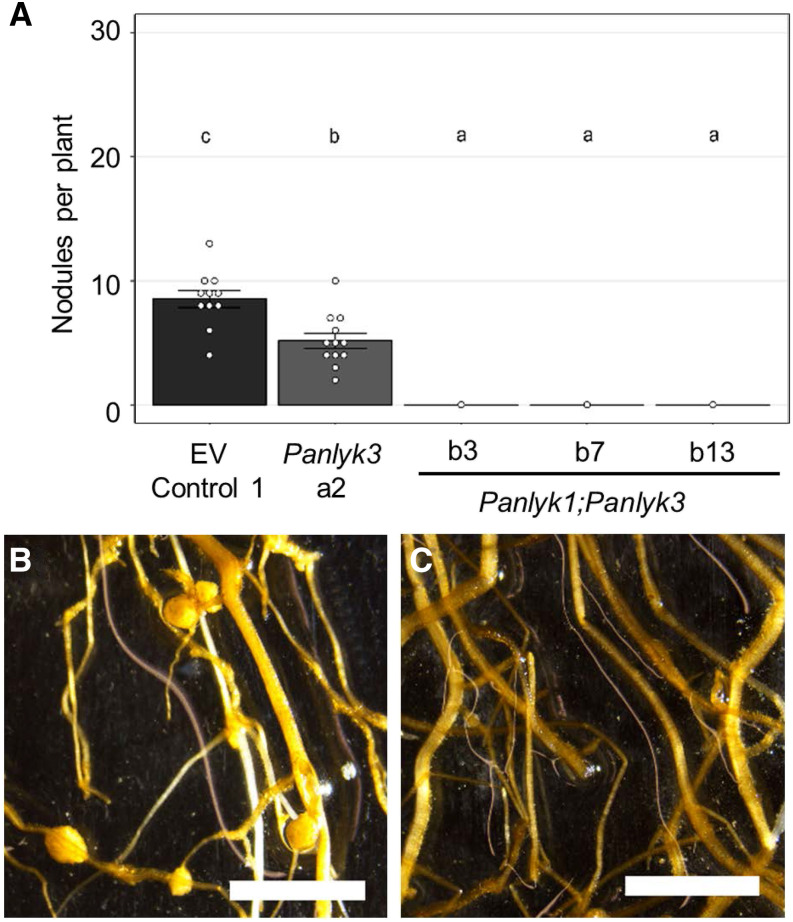
*P. andersonii PanLYK1* and *PanLYK3* act redundantly in nodulation. A, Average nodule numbers per plant in EV Control 1 (*n* = 11) and retransformed *Panlyk3* line a2 (*n* = 12) and *Panlyk1;Panlyk3* double mutant lines b3 (*n* = 10), b7 (*n* = 5), and b13 (*n* = 10) at 5 wpi with *M. plurifarium* BOR2. Data are represented as means ± se, and circles represent individual data points. Lowercase letters denote statistical significance based on one-way ANOVA and Tukey’s posthoc contrast (*P* > 0.05). B, Roots with nodules of EV Control 1 at 5 wpi with *M. plurifarium* BOR2. C, Roots without nodules of the *Panlyk1;Panlyk3* double mutant (line b3) at 5 wpi with *M. plurifarium* BOR2. Bars = 5 mm.

As *P. andersonii* did not experience any gene duplication events in the LYK-Ib clade, *PanLYK1* in the LYK-Ia clade is the closest homolog of *PanLYK3*. In order to investigate whether the *PanLYK1* gene is functionally redundant with *PanLYK3* in cases of *M. plurifarium* BOR2 inoculation, we generated a *Panlyk1;Panlyk3* double mutant. To do so, a binary construct with the two sgRNAs targeting *PanLYK1* was used for retransformation of the *Panlyk3* mutant (line a2). We obtained three independent *Panlyk1;Panlyk3* mutants (Supplemental Data Set S3). *M. plurifarium* BOR2 inoculation experiments revealed that all *Panlyk1;Panlyk3* double mutant lines were unable to form any nodule or nodule-like structure (Fig. 6). To confirm that the nodulation-minus phenotype in the *Panlyk1;Panlyk3* lines is not due to any off-target mutation, we conducted complementation studies using *A. rhizogenes*-mediated root transformation. As the putative promoter of *PanLYK3* is rather complex due to the occurrence of alternative transcriptional start sites (Fig. 2), we used the *LjNFR1* promoter, as well as the constitutive *AtUBQ10* and *CaMV35S* promoters, to drive a CRISPR-resistant allele of *PanLYK3.1* (*PanLYK3cr*). Compound plants carrying transgenic roots expressing *PanLYK3cr* could be nodulated by *M. plurifarium* BOR2 (Supplemental Fig. S5). Together, this showed that in *P. andersonii*, *PanLYK1* and *PanLYK3* act redundantly in root nodule formation. (For complementation studies of *Pannf2*, see below.)

The results demonstrate that *P. andersonii PanLYK1*, *PanLYK3*, *PanNFP1*, and *PanNFP2* function in rhizobium LCO-driven nodulation. *PanLYK3* and *PanNFP2* are orthologous to legume *LjNFR1/MtLYK3* and *LjNFR5/MtNFP*, indicating a shared evolutionary origin of LCO-driven nodulation in both taxonomic lineages. As *PanLYK1* and *PanLYK3* evolved from a duplication predating the emergence of the nitrogen-fixing clade, this suggests that LCO signaling is an ancestral function of these LYK-I receptors.

### A Repaired *Trema levigata NFP2* Pseudogene, But Not *PanNFP1,* Can Functionally Complement a *P. andersonii nfp2* Mutant

**Figure 7.**
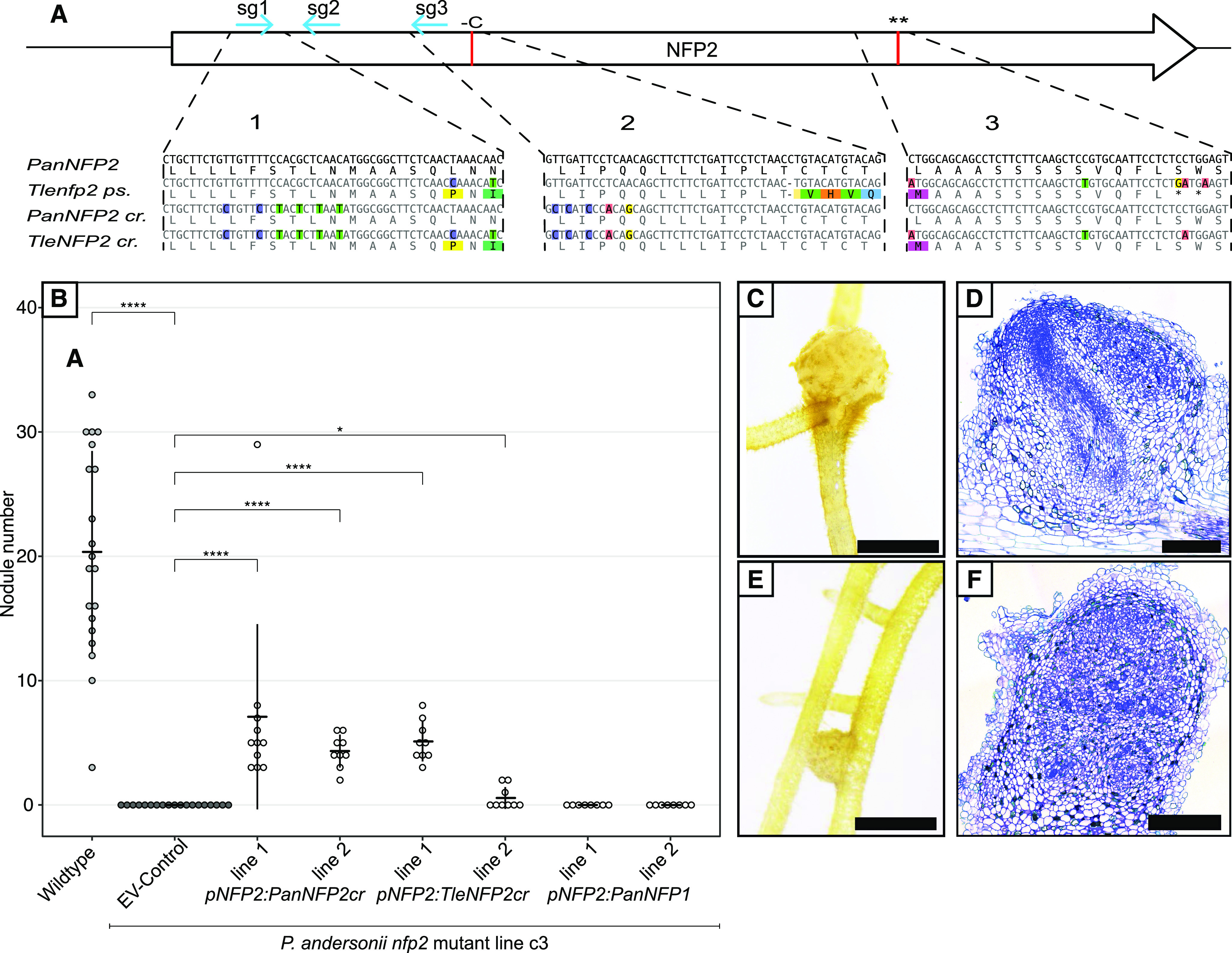
A repaired *T. levigata nfp2* pseudogene can replace *PanNFP2* for nodule formation. A, Schematic representation of the NFP2 coding region with indicated replacements to avoid CRISPR targeting of inserted *NFP2* genes of *P. andersonii* (*PanNFP2cr*) and a repaired *T. levigata* (*TleNFP2cr*). Blue arrows indicate guide RNA target sites, and red lines indicate *T. levigata* mutations. Region 1, Replacement of six codons at the sg1 site; region 2, replacement of five codons at the sg3 site plus repair of the *T. levigata* indel (red line); region 3, repair of the double stop codon in *T. levigata* (red line, black asterisks). The replacement of five codons at the sg2 site is not shown. B, *PanNFP2cr* and repaired *TleNFP2cr* can restore nodulation in the *Pannfp2* mutant line C3 when driven by the *PanNFP2* promoter, whereas *PanNFP1* cannot. Nodulation was scored at 5 wpi with *M. plurifarium* BOR2. Error bars represent the sd. Asteriks indicate statistical significance by Mann-Whitney-Wilcoxon test (*P < 0.05 and ****P < 0.00001). C and D, Nodule and section of *pNFP2:PanNFP2cr* line 1. E and F, Nodule and section of *pNFP2:TleNFP2cr* line 1. Bars = 2 mm (C and E) and 100 μm (D and F).

*PanNFP1* and *PanNFP2* differ in expression pattern. Whereas both genes are expressed in root tissue, only *PanNFP2* is up-regulated in nodules (Fig. 2B; van Velzen et al., 2018). We questioned whether the difference in symbiotic functioning between both genes is the result of regulatory evolution. To test this, we first identified a functional promoter region of *PanNFP2*. *A. tumefaciens*-mediated transformation showed that a 2.75-kb *PanNFP2* upstream region can be used to functionally complement the *P. andersonii Pannfp2* mutant when using a *PanNFP2* CRISPR-resistant allele (*PanNFP2cr*). Two independent lines formed 7 ± 7 and 4 ± 1 nodules 5 wpi with *M. plurifarium* BOR2 (Fig. 7). However, when we used *PanNFP1* driven by the *PanNFP2* promoter, no transcomplementation of the *P. andersonii nfp2* mutant phenotype was observed. This suggests that there is a functional difference in the encoded *PanNFP1* and *PanNFP2* receptors.

Next, we questioned whether the *nfp2* pseudogene as present in several nonnodulating Rosales species may have encoded a functional symbiosis receptor. To test this, we focused on the *nfp2* pseudogene of *T. levigata*, as it has only three mutations that cause a disturbance of the open reading frame (Fig. 7). We repaired these three mutations, using *PanNFP2* as a template, resulting in an engineered CRISPR-resistant *TleNFP2cr* that encodes for a LysM-type receptor protein of 582 amino acids, similar to *PanNFP2* of *P. andersonii*. We tested whether *TleNFP2cr* driven by the *PanNFP2* promoter can transcomplement the *P. andersonii Pannfp2* mutant. *A. tumefaciens* transformation resulted in two lines that can form functional root nodules 5 wpi with *M. plurifarium* BOR2. This supports the hypothesis that *T. levigata nfp2* encoded a functional symbiosis receptor prior to the pseudogenization of this gene.

### *P. andersonii PanLYK3* Is Essential for Chitin-Triggered Immune Responses and Controls AM Symbiosis in Coherence with *PanLYK1*

Next, we aimed to determine whether the *P. andersonii* LysM-type receptors that control nodulation are also involved in other processes, as this may provide insights into ancestral functions of these genes. Some LysM-type receptors of the LYK-I clade are known to function in chitin-triggered immunity and/or the AM symbiosis: *L. japonicus* LjCERK6, *M. truncatula* MtLYK9/MtCERK1, Arabidopsis AtCERK1, tomato SlLYK1, and rice OsCERK1 (Fig. 1; Miya et al., 2007; Wan et al., 2008; Shimizu et al., 2010; Bozsoki et al., 2017; Liao et al., 2018; Feng et al., 2019; Gibelin-Viala et al., 2019; He et al., 2019). Similarly, some experimental evidence using transient silencing assays indicated that LysM-type receptors of the LYR-IA clade function in mycorrhization, including *P. andersonii* PanNFP1 (Op den Camp et al., 2011). In line with this, we aimed to confirm this phenotype in stable *Pannfp1* knockout mutants and determine whether other *P. andersonii* symbiotic LysM-type receptors may also function in AM symbiosis and/or chitin-induced innate immunity signaling.

**Figure 8.**
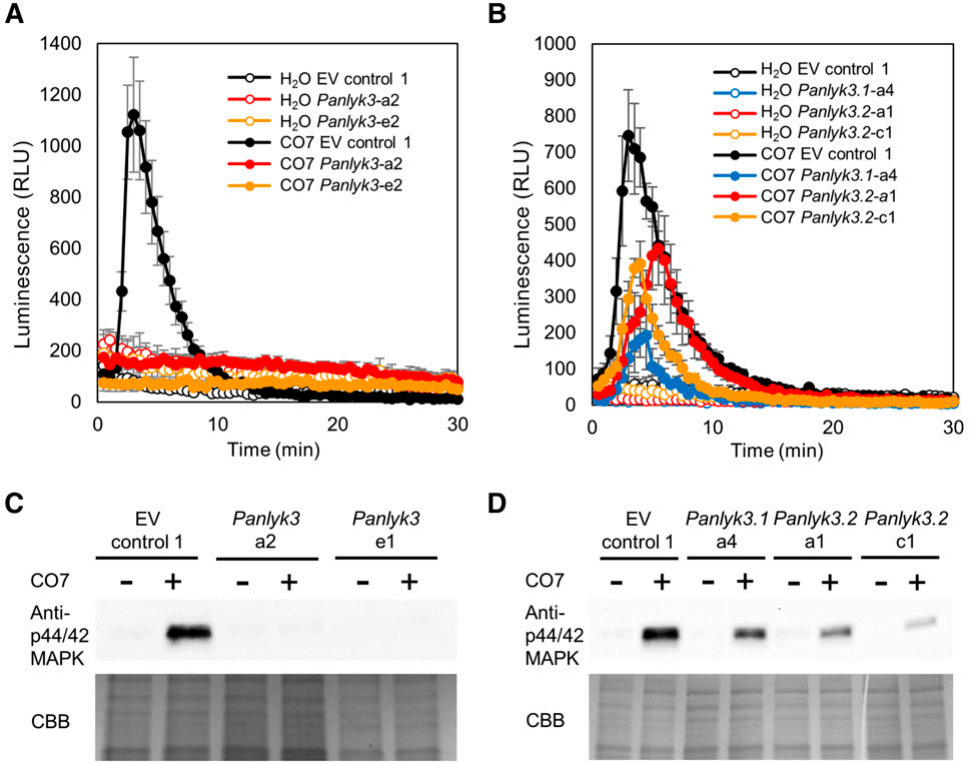
*P. andersonii PanLYK3* is essential for chitin-triggered immunity responses in roots. A and B, Production of ROS measured upon treatment with 100 μm CO7 (filled circles) or water (open circles) with EV Control 1 plants (black), *Panlyk3* line a2 (red), and *Panlyk3* line e1 (orange; A) and EV Control 1 plants (black), *Panlyk3.1* line a4 (blue), *Panlyk3.2* line a1 (red), and *Panlyk3.2* line c1 (orange; B). For A and B, data are averages of at least three independent biological replicates ± se. Luminescence is measured in relative light units (RLU). C and D, Phosphorylation of MAPK analyzed by immunoblot using an anti-p44/42 MAPK antibody upon treatment with 100 μm CO7 (top). Equal loading was confirmed by Coomassie Brilliant Blue (CBB) staining (bottom). Results shown are representative of three independent experiments. C, MAPK phosphorylation in root pieces of EV Control 1, *Panlyk3* line a2, and *Panlyk3* line e1. D, MAPK phosphorylation in root pieces of EV Control 1, *Panlyk3.1* line a4, *Panlyk3.2* line a1, and *Panlyk3.2* line c1.

First, we investigated whether the *P. andersonii* LysM-type receptor mutants are affected in chitin-triggered immunity responses. To do so, two complementary assays were used: a chitin-induced reactive oxygen species (ROS)-burst production and a MITOGEN-ACTIVATED PROTEIN KINASE3 (MAPK3)/MAPK6 phosphorylation assay. Chitin heptamers (CO7) effectively induced a ROS burst in *P. andersonii* root segments at concentrations of 1 μm or greater when incubated at 28°C, the regular growth temperature of *Parasponia* spp. (Fig. 8A; Supplemental Fig. S6B). To test whether ROS bursts can also be triggered by rhizobium LCOs, we used the extracts of *M. loti* R7A and *Rhizobium tropici* CIAT899. These two strains can nodulate *P. andersonii* but produce structurally different LCOs (López-Lara et al., 1995; Folch-Mallol et al., 1996). However, neither triggered a ROS burst in *P. andersonii* roots (Supplemental Fig. S6A). To determine whether CO7-induced ROS bursts were associated with phosphorylation of *P. andersonii* MAPK3 and MAPK6 homologs, we used an anti-phospho-p44/42 HsMAPK antibody, which detects phosphorylated MAPK3 and MAPK6 of different plant species (Yamaguchi et al., 2013; Bozsoki et al., 2017). *P. andersonii* possesses a single *PanMAPK3* gene and a single *PanMAPK6* gene, each of which encodes a protein with a conserved Thr-202/Tyr-204 phosphorylation site (Supplemental Fig. S6C). Upon CO7 application (100 μm, 10 min), a MAPK3/6 phosphorylation pattern can be detected, which is not observed upon application of *M. loti* or *R. tropici* LCO extracts (Fig. 8C; Supplemental Fig. S6D). Next, we determined whether *P. andersonii* LysM-type receptor mutants are affected in response to chitin CO7 oligomers. *Pannfp1*, *Pannfp2*, and also a newly created *Pannfp1;Pannfp2* double mutant showed a wild-type ROS-burst and MAPK3/6 phosphorylation profile (Supplemental Fig. S6; Supplemental Data Set S3). Similarly, the *Panlyk1* mutant showed a ROS-burst and MAPK3/6 phosphorylation profile, as did wild-type root segments (Supplemental Fig. S6, E and F). In contrast, *P. andersonii Panlyk3* mutant lines lacked a chitin-triggered ROS burst and showed no p44/42 MAPK phosphorylation (Fig. 8). Individual exon-knockout *Panlyk3.1* or *Panlyk3.2* mutants both showed ROS production and MAPK3/6 phosphorylation upon application of 100 μm CO7, although at reduced levels (Fig. 8, B and D). Taken together, these data show that *PanLYK3*, which is the only *P. andersonii* gene in the LYK-Ib clade, is essential for chitin innate immune signaling in roots.

**Figure 9.**
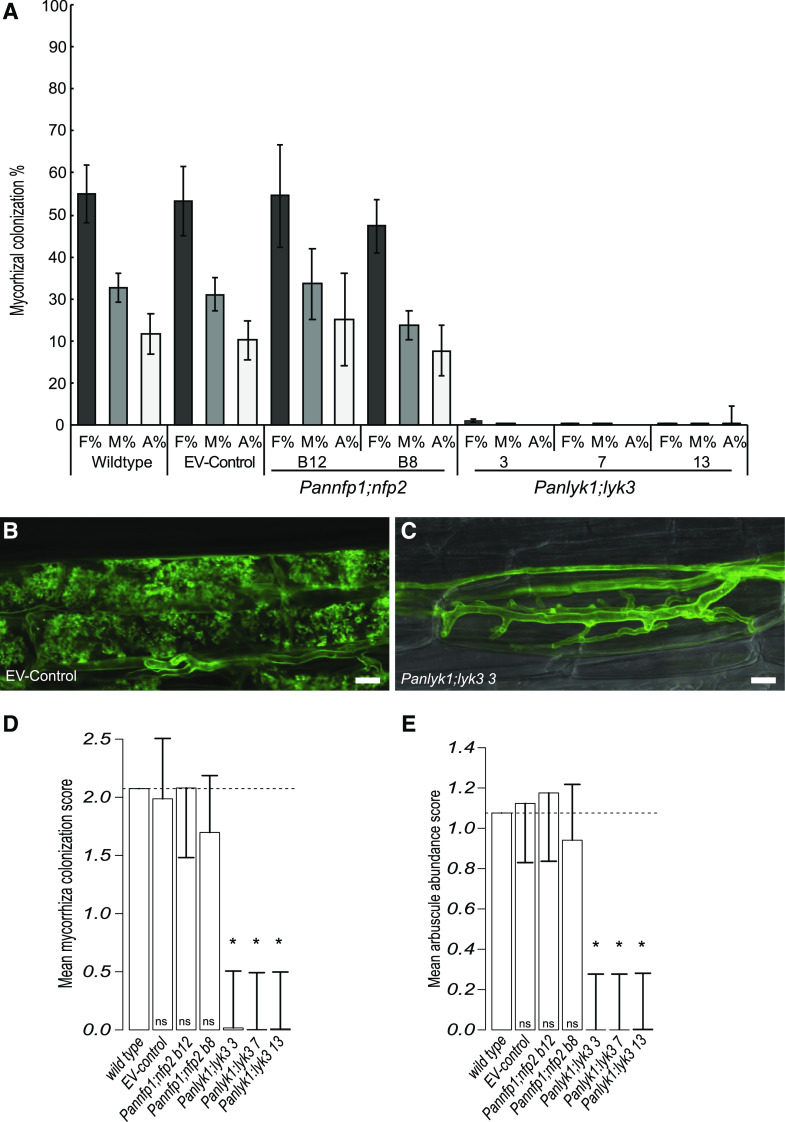
*P. andersonii PanLYK1* and *PanLYK3* act redundantly in arbuscular mycorrhization. A, The *P. andersonii Panlyk1;Panlyk3* double mutant shows a strongly reduced colonization compared with wild-type and control *P. andersonii* roots. *P. andersonii Pannfp1;Pannfp2* mutants are not significantly affected. Frequency and arbuscule abundance classes are according to Trouvelot et al. (1986). F%, Colonization frequency in the root system; M%, intensity of mycorrhizal colonization; A%, arbuscule abundance in the root system. Error bars represent the se of 10 biological replicates scored at 6 wpi using 250 spores of *R. irregularis* strain DOAM197198 (Trouvelot et al., 1986). B, Highly branched arbuscules formed in EV control plants at 6 wpi stained with WGA-Alexa488. C, Phenotype of stunted arbuscules formed in the *Panlyk1;Panlyk3* double mutant stained with WGA-Alexa488. Bars = 10 μm. D and E, Statistical analysis of raw (observed) data: mean colonization frequency score (classes 0–5; D) and mean arbuscule score (classes 0–3; E). Classes are presented according to Trouvelot et al. (1986). Reduced mycorrhizal colonization and arbuscule formation in *Panlyk1;Panlyk3* mutants are considered significant compared with the wild type. Error bars represent the Bonferroni-corrected lsd. Error bars nonoverlapping with the mean wild-type value are considered significant. Dashed lines indicate the mean wild-type score. Asterisks mark samples that are significantly different for wild type.

Studies in *P. andersonii*, tomato, *M. truncatula*, and rice revealed that LYR-IA and LYK-I putative orthologous genes have functions in AM symbiosis (Miyata et al., 2014, 2016; Zhang et al., 2015; Buendia et al., 2016; Carotenuto et al., 2017; Liao et al., 2018; Feng et al., 2019; Gibelin-Viala et al., 2019; He et al., 2019). Interestingly, we noted that the NFP-I-type gene is pseudogenized in European beech and Chinese chestnut. Both species have lost AM symbiosis in favor of an ectomycorrhizal symbiosis (Fig. 3; Werner et al., 2018). We conducted an RNA sequencing experiment on *P. andersonii* roots mycorrhized by *Rhizophagus irregularis* strain DOAM197198. Several marker genes for mycorrhization were shown to be enhanced in expression in mycorrhized *P. andersonii* root samples, including *PanSTR1*, *PanSTR2*, *PanPT4*, *PanVPY*, *PanD27*, *PanRAD1*, and *PanRAM1* (Supplemental Fig. S7). Also, this suggested that *PanNFP1* is expressed at a higher level than *PanNFP2* under these conditions (Supplemental Fig. S7). However, no significant differential regulation of any of the studied LysM-type receptor-encoding genes was detected between phosphate-starved control roots and mycorrhized root samples (Supplemental Fig. S7). To determine whether *P. andersonii* symbiotic LysM-type receptors also function in AM symbiosis, we conducted three independent experiments using in vitro-propagated mutant plantlets inoculated with 250 spores of *R. irregularis* DOAM197198. The average colonization and arbuscule formation frequency were scored 6 wpi. These experiments revealed substantial variation in mycorrhization efficiency between replicates, although no clear impaired AM symbiosis phenotype could be observed in any of the single mutants, including *Pannfp1*. Strikingly, *Panlyk1* showed a significant increase in colonization and arbuscule frequency (Supplemental Fig. S8, A–C). Analyzing both double mutants, *Pannfp1;Pannfp2* and *Panlyk1;Panlyk3*, revealed a strong AM symbiosis phenotype only in the latter (Fig. 9; Supplemental Fig. S8). The fungal colonization of the *Panlyk1;Panlyk3* mutant was severely affected, with only a few infections observed. Confocal imaging of wheat germ agglutinin conjugated to Alexa Fluor 488 (WGA-Alexa488)-stained roots showed that besides the level of colonization, also the morphology of the few arbuscules that were formed was affected in *Panlyk1;Panlyk3* plants. In wild-type plants, many cortical cells were filled with arbuscules that were finely branched and occupied most of the cell. In contrast, the few hyphae that enter cortical cells in the *Panlyk1;Panlyk3* mutant were unable to form mature arbuscules, either because the fungus fails to switch to fine branching or because a limited number of fine branches is made (Fig. 9). As both *Panlyk1* and *Panlyk3* single mutant plants do not show this impaired mycorrhizal phenotype, we conclude that both genes function in conjunction to control mycorrhizal infection.

Taken together, these experiments revealed that PanLYK1 and PanLYK3 can function in multiple processes, including rhizobium nodulation (PanLYK1 and PanLYK3), AM symbiosis (PanLYK1 and PanLYK3), and chitin innate immune signaling (PanLYK3). This suggests that no subfunctionalization of these receptors is required to allow functioning in the rhizobium nitrogen-fixing nodulation trait.

## DISCUSSION

We used *P. andersonii* as a comparative system to legumes to obtain insight into the evolutionary trajectory of LysM-type rhizobium LCO receptors. By conducting phylogenetic analysis, transcomplementation studies in a *L. japonicus* LCO receptor double mutant, and CRISPR-Cas9 mutagenesis in *P. andersonii*, we identified four LysM-type receptors that function in LCO-driven nodulation in a nonlegume. Two of these, *PanLYK3* and *PanNFP2*, are putative orthologs to known legume rhizobium LCO receptors *LjNFR1/MtLYK3* and *LjNFR5/MtNFP*, respectively. As the *Parasponia* spp. and legume lineages diverged early in the nitrogen-fixing clade (more than 100 million years ago), the use of orthologous genes for rhizobium LCO perception supports the hypothesis of a shared evolutionary origin of LCO-driven nodulation. In contrast to legumes, symbiotic LysM-type receptors in the genus *Parasponia* did not experience recent duplication events. Instead, the *Parasponia* spp. symbiotic LysM-type LCO receptors evolved following two ancient duplications. We hypothesize that the *PanNFP1*-*PanNFP2* duplication associates with the origin of the nitrogen-fixing clade, whereas in the case of *PanLYK1* and *PanLYK3*, the duplication occurred prior to the birth of the nitrogen-fixing clade. This makes it most probable that the capability of these receptors to perceive LCOs predates the evolution of the nitrogen-fixing nodulation trait.

Presently, the *NFP1-NFP2* duplication cannot be precisely dated because legumes do not possess an NFP-I-type gene. This can be explained in two scenarios. (1) The *NFP1-NFP2* duplication occurred in the root of the nitrogen-fixing clade, and subsequently, the NFP-I-type gene got lost in the Fabales lineage. (2) The *NFP1-NFP2* duplication occurred in an ancestor of the Fagales-Cucurbitales-Rosales lineages after the divergence of the Fabales order. The recent finding that ectopic expression of the *NFP*-type gene of two species outside of the nitrogen-fixing clade (*Petunia hybrida PhLYK10* and tomato *SlLYK10*) can, at least partially, transcomplement the *M. truncatula* and *L. japonicus* *Mtnfp* and *Ljnfr5* mutant phenotypes demonstrates that LCO receptor functionality is ancestral to the *NFP1-NFP2* duplication (Girardin et al., 2019). The putative promoters of *PhLYK10* and *SlLYK10* show a nodule-enhanced expression profile similar to that reported for *PanNFP2* (Girardin et al., 2019), which may support the second scenario, where the duplication of *NFP1-NFP2* occurred only after the divergence of the Fabales clade. However, for such a scenario, it is essential that Fabales represents the most basal lineage in the nitrogen-fixing clade. To date, this remains unknown. For example, a recent phylogenetic study suggests, although with limited statistical support, that Fabales is sister to Fagales (Koenen et al., 2020). The phylogenetic analysis presented here (Fig. 3) suggests that the first scenario is most probable (aSH-aLRT/UF-Bootstrap/approximation with Mr.Bayes support 76.4/77/0.859). Additionally, we searched for amino acid motifs in NFP-I- and NFP-II-type proteins and found an indel region in legume and nonlegume NFP-II-type proteins that is distinct from NFP-I (Supplemental Fig. S9). This also supports the hypothesis that NFP1-NFP2 duplicated at the root of the nitrogen-fixing clade. However, additional experiments are needed to definitively reject either scenario.

Transcomplementation studies in a *L. japonicus* *Ljnfr1;Ljnfr5* double mutant showed that *P. andersonii* LCO receptors can only partially restore LCO signaling. This only partial complementation we did not anticipate, because of the shared microsymbiont *M. loti* that can nodulate *P. andersonii* well as *L. japonicus*. One explanation for this limited functionality may be that such receptors function in larger multiprotein membrane domain complexes. In such a case, the *P. andersonii* LCO receptors are not adapted to interact with associated *L. japonicus* proteins. Additionally, legumes and *Parasponia* spp. have diverged in the mode of rhizobium infection. Whereas rhizobium penetrates *Parasponia* spp. roots apoplasticly by crack entry, legumes are generally infected intracellularly via curled root hair cells. Phenotypic analysis of rhizobium infection in legumes suggests that a specific LCO receptor is involved in this process, the so-called entry receptor (Ardourel et al., 1994). Such entry receptors have not yet been fully characterized, but *MtLYK3* may carry out such functions, as they control rhizobium infection (Limpens et al., 2003; Smit et al., 2007). It remains elusive whether such entry receptor functioning requires specific adaptations that did not occur in the genus *Parasponia LYK3* ortholog.

We showed that an engineered *TleNFP2* receptor can functionally complement the *P. andersonii Pannfp2* mutant, whereas *PanNFP1* cannot. This suggests that the NFP1 and NFP2 receptor proteins have functionally diverged. Based on the finding that NFP-orthologous proteins of *P. hybrida* (PhLYK10) and tomato (SlLYK10) can complement *L. japonicus* *Ljnfr5* and *M. truncatula Mtnfp* mutants, it can be hypothesized that, in *P. andersonii* especially, *PanNFP1* has experienced protein adaptations. However, it should be noted that the transcomplementation studies presented here were conducted using the native *PanNFP2* promoter, whereas studies conducted with *PhLYK10* and *SlLYK10* were conducted with *CAMV35S* (Girardin et al., 2019). Such overexpression may mask differences in substrate affinity and/or specificity, under which native transcriptional regulation is biologically relevant. Our data demonstrate that the ancestor of *T. levigata* possessed an NFP2 receptor that can function in nodulation.

Mutant analysis in legumes demonstrated that rhizobium nodulation coopted elements of an AM signaling pathway, including the Leucine-rich repeat-type transmembrane receptor kinase *L. japonicus* SYMBIOTIC RECEPTOR KINASE/ *M. truncatula* DOES NOT MAKE INFECTIONS2 (MtDMI2), the nuclear envelope-located cation ion channels LjCASTOR, LjPOLLUX/MtDMI1, the nucleus-localized CALCIUM CALMODULIN KINASE LjCCaMK/MtDMI3, and the transcription factor LjCYCLOPS/*M. truncatula* INTERACTING PROTEIN OF DMI3 (Geurts et al., 2012). However, in legumes, rhizobium and AM fungi were shown to have independent perception mechanisms to activate this common symbiosis signaling pathway. In *L. japonicus* and *M. truncatula*, these consist of LjNFR1-LjNFR5/MtLYK3-MtNFP for rhizobium LCOs and MtLYK9/MtCERK1 for AM signals (Geurts et al., 2012; Feng et al., 2019; Gibelin-Viala et al., 2019). MtLYK3 and MtLYK9/MtCERK1 both belong to the LYK-Ib clade and evolved following legume-specific duplication events (Fig. 1; De Mita et al., 2014). The strong phenotype in AM and nodule symbioses of the *P. andersonii Panlyk1;Panlyk3* knockout mutant demonstrates that such subfunctionalization is not causal for the evolution of rhizobium LCO receptors. In *P. andersonii*, both receptors function in conjunction to control AM and rhizobium nodulation. Additionally, PanLYK3 acts as a chitin innate immune receptor. Such multifunctionality has also been reported for MtLYK9/MtCERK1 in *M. truncatula* and OsCERK1 in rice, which function both in AM symbiosis and chitin innate immune signaling (Miyata et al., 2014; Carotenuto et al., 2017; Feng et al., 2019; Gibelin-Viala et al., 2019). As monocots did not experience the LYK-Ia/LYK-Ib duplication, this demonstrates that performing multiple functions in symbioses and innate immunity was ancestral to species of the nitrogen-fixing clade but functionally diverted in the legume lineage.

The presence of NFP-type genes (LYR-IA orthogroup) in species outside of the nitrogen-fixing clade is associated with the ability to establish an AM symbiosis (Fig. 3; Delaux et al., 2014; Gough et al., 2018). However, corresponding mutants have only a relatively weak phenotype in AM symbiosis (Buendia et al., 2016; Miyata et al., 2016; Girardin et al., 2019). Upon duplication of this gene, the NFP-I and NFP-II subclades may have inherited the ancestral function. As both the *P. andersonii* PanNFP1 and PanNFP2 receptors can partially complement LCO-induced Ca^2+^ oscillation in the *L. japonicus* *Ljnfr1;Ljnfr5* double mutant, this supports that receptors of the NFP-I and NFP-II clades can act as an LCO receptor, which may reflect the ancestral function. Our observation that the presence of a functional gene in the NFP-II clade strictly associates with LCO-based nodulation suggests that this gene was coopted to function in this trait. The importance of this LysM-type LCO receptor in the nitrogen-fixing nodulation trait is underlined by the complete block of nodulation in knockout mutants in legumes (e.g. *L. japonicus* *Ljnfr5*, *M. truncatula Mtnfp*, and pea *Pssym10*) and the genus *Parasponia* (*P. andersonii Pannfp2*; Madsen et al., 2003; Arrighi et al., 2006). As the genus *Parasponia* and legumes diverged at the root of the nitrogen-fixing clade, this suggests that the adaptations in the NFP-II clade are ancient and may have coincided with the birth of the nodulation trait.

The NFP-I-type gene retained, at least in part, its ancestral function, indicated by its presence in nonnodulating species in the nitrogen-fixing clade that can establish an AM symbiosis. In cases where AM symbiosis is replaced by an ectomycorrhizal symbiosis, such as in European beech or Chinese chestnut*,* the NFP-I-type gene pseudogenized. However, phenotypic studies in stable *P. andersonii* mutants could not support the functioning of *PanNFP1* in AM symbiosis. These findings contradict our earlier observation that this gene functions in arbuscule formation (Op den Camp et al., 2011). The reason for this discrepancy may be due to the RNAi construct used, which may have off-target effects (van Velzen et al., 2018). To determine whether this is the case, we have studied the expression of LysM-type RLK genes in two independent *PanNFP1* RNAi experiments. This revealed significant knockdown not only of *PanNFP1* but also *PanNFP2*, which can explain the strong rhizobium nodulation and infection phenotype as reported by Op den Camp et al. (2011). We also found variable expression levels of other LysM-type RLKs, including *PanLYK1* and *PanLYK3*, which may explain the reported mycorrhization phenotype on *PanNFP1* RNAi roots (Supplemental Fig. S10). The studies presented here using CRISPR-Cas9 knockout mutant lines revealed substantial biological variation in mycorrhization efficiency of *P. andersonii* roots, which may have hindered the observation of minor quantitative AM symbiosis phenotypes. To rule out that PanNFP1 and PanNFP2 may function redundantly to control AM symbiosis, we analyzed a *Pannfp1;Pannfp2* double mutant. Also, these lines were shown to be effectively mycorrhized. Therefore, we conclude that our current mutant phenotype analysis does not find support for essential functioning of *P. andersonii* PanNFP1 and PanNFP2 in AM symbiosis.

The study presented here provides insight into the evolutionary trajectory of symbiotic LCO LysM-type receptors. By using *P. andersonii* as a comparative system to legumes, we revealed two ancestral duplications of LysM-type LCO receptors that predate, and coincide with, the evolution of nitrogen-fixing nodules. The strict association of genes in the NFP-II clade with LCO-driven nodulation strongly suggests that this gene was coopted to function specifically in this symbiosis, making NFP2 a target in approaches to engineer LCO-driven nodulation in nonleguminous plants.

## MATERIALS AND METHODS

### LysM-Type Receptor Phylogeny Reconstructions

Orthogroups containing LysM-type receptor kinases of *Parasponia andersonii*, generated in a previous study (van Velzen et al., 2018), were combined and realigned into a single alignment using MAFFTV7.017 (Kazutaka and Schandley, 2013). MrBayes3.2.6 (Ronquist and Huelsenbeck, 2003; Ronquist et al., 2012) was used to calculate phylogenetic relations under default parameters in Geneious R8.1.9 (Biomatters ). Clades were named as published previously (Buendia et al., 2018). For clades LYK-I and LYR-IA, additional putative orthologs were collected from the Phytozome and National Center for Biotechnology Information databases using BLAST with AtCERK and MtNFP protein sequences as queries (Supplemental Table S1; Altschul et al., 1997). Available genomes from Fabales, Fagales, Cucurbitales, and Rosales species were downloaded, and local BLAST analysis was conducted using Geneious R8.1.9 (Biomatters) to search for additional unannotated LYK-I and LYR-IA protein sequences. Pseudogenes were annotated manually based on the closest functional ortholog so that a protein sequence could be deduced. Correct protein sequences were aligned using MAFFTV7.017 and subsequently manually curated. The deduced amino acid sequence was subsequently added to the alignment if the alignment length was at least 70% of the *P. andersonii* protein. Phylogenetic analysis was performed using IQ-tree (Nguyen et al., 2015; Trifinopoulos et al., 2016), running the modelfinder extension to find the best substitution models (Kalyaanamoorthy et al., 2017). Branch support analysis was done using Sh.aLRT 1,000 replicates, UF-BOOTSTRAP support 1,000 iterations (Kalyaanamoorthy et al., 2017; Hoang et al., 2018), and approximate Bayes support. Branch supports shown are UF-Bootstrap support%. The best fit model for the LYK-I clade was JTT+I+G4, and the best fit model for the LYR-IA clade was JTT+I+G4. Resulting tree files were loaded into Interactive Tree Of Life v3 for editing (Letunic and Bork, 2016). The analysis was run at least three times. Trees were rooted to the outgroup angiosperm species *Amborella trichopoda*. UF Bootstrap Branch supports >98 were omitted for visual clarity. Gene names, accession numbers, and alignment files of identified homologs can be found in Supplemental Data Set S1 for LYK-I, Supplemental Data Set S2 for LYR-IA, and Supplemental Table S1 for *P. andersonii*.

### LYK3 Alignment and Variant Detection

Genomic LYK3 regions of *P. andersonii*, *Parasponia rigida*, *Parasponia rugosa*, *Trema orientalis* RG16, *T. orientalis* RG33, and *Trema levigata* were extracted from the respective assemblies (van Velzen et al., 2018) and aligned using MAFFTV7.0.17 implemented in Geneious R8.1. Coding sequences of *P. andersonii*, *P. rigida*, and *P. rugosa* LYK3 protein variants were translated and aligned using MAFFTV7.0.17 implemented in Geneious R8.1 (Supplemental Data Set S1).

### Vector Constructs

All vectors generated for this study were created using golden gate cloning (Engler et al., 2009). Backbones and binary vectors were derived from the golden gate molecular toolbox (Engler et al., 2014). *P. andersonii* LysM-type receptor cDNA clones were sequence synthesized as level 0 modules, including silent mutations in golden gate *Bsa*I or *Bpi*I restriction sites. Golden gate-compatible clones of LjNFR1 and LjNFR5 promoters, coding sequences, and terminators were obtained from Aarhus University. The calcium signaling reporter pLjUBQ1:R-GECO1.2 was published previously (Kelner et al., 2018). The generation and assembly of *P. andersonii* CRISPR constructs were done as published previously (van Zeijl et al., 2018). For hairy root transformation, a modified level 2 standard vector carrying spectinomycin instead of kanamycin resistance was created. All sgRNAs were expressed using the AtU6 promoter. All golden gate binary vectors were verified by restriction digestion and DNA sequencing before transformation. Lists of primers and constructs can be found in Supplemental Tables S3 and S4.

### Genotyping and Off-Target Analysis

All sgRNA targets were designed using the Geneious R10 CRISPR design tool, which picks targets on the principles described by Doench et al. (2014). To be selected, guide RNAs must have no potential target sites in the genome with (1) less than three mismatches or (2) less than two indels. Known off-target locations in coding sequence regions were PCR amplified and sequenced. No off-target mutations at these sites were detected. Genotypes and known off-target locations of CRISPR mutants used in this study can be found in Supplemental Data Set S3. Primers used for the creation of sgRNAs and subsequent sequencing of mutants and off-targets are listed in Supplemental Table S4.

### Bacterial Strains

We used *Mesorhizobium plurifarium* BOR2 (van Velzen et al., 2018) and *Sinorhizobium fredii* NGR234.pHC60 expressing GFP (Trinick and Galbraith, 1980; Cheng and Walker, 1998; Op den Camp et al., 2011) for *P. andersonii* inoculation experiments. *Mesorhizobium loti* R7A.pHC60 (Cheng and Walker, 1998; Sullivan et al., 2002) was used for *Lotus japonicus* inoculations. *M. loti* R7A and *Rhizobium tropici* CIAT899 (Martínez-Romero et al., 1991) containing plasmid pMP604 (Spaink et al., 1989) were used for LCO extraction. *Agrobacterium rhizogenes* strain AR10 (Hansen et al., 1989; Martínez-Romero et al., 1991) was used for *L. japonicus* root transformation. *Agrobacterium tumefaciens* strain AGL-1 (Lazo et al., 1991) was used in *P. andersonii* transformation. *Agrobacterium* sp. MSU440 was used for *P. andersonii* hairy root transformation (Cao et al., 2012). The *Escherichia coli* strain DH5α was used to propagate plasmids and in all subsequent cloning steps.

### Rhizobium LCO Isolation

To isolate rhizobium LCOs, the plasmid pMP604 containing an autoactive NodD protein was introduced in *M. loti* R7A and *R. tropici* CIAT899 (Spaink et al., 1989; López-Lara et al., 1995). LCOs were extracted from a 750-mL liquid culture, OD_600_ = 0.5, grown at 28°C in minimal medium (5.75 mm K_2_HPO_4_, 7.35 mm KH_2_PO_4_, 5.9 mm KNO_3_, 460 nm CaCl_2_, 37.5 µm FeCl_3_, 2.07 mm MgSO_4_, 20.5 nm biotin, 2.9 nm thiamine-HCl, 8.1 nm nicotinic acid, 4.8 nm pyridoxine-HCl, 2.8 nm myoinositol, 4.6 nm panthotenate, and 1% [w/v] Suc) by the addition of 150 mL of 1-butanol and 1 h of shaking. The butanol phase was transferred and subsequently evaporated (water bath at 40°C). The pellet was dissolved in 75 mL of methanol, tested for Nod factor activity, and stored at −20°C for later use. The concentration of active LCOs was estimated by using *LjNIN* induction in *L. japonicus* wild-type cv Gifu roots 3 h postapplication. The lowest active dilution was estimated to be ∼10^−10^ m.

### *L*.* japonicus A. rhizogenes* Root Transformation

*L. japonicus* *Ljnfr1-1;Ljnfr5-2* double mutants (Madsen et al., 2003; Radutoiu et al., 2003) were used for LysM complementation assays and cv Gifu wild type as control. Seedlings for *A. rhizogenes* root transformation were moved to fresh one-half-strength B5 medium and cocultivated for 1 week as described previously using *A. rhizogenes* strain AR10 (Stougaard et al., 1987; Hansen et al., 1989; Stougaard, 1995). During root emergence, plants were grown on 1% (w/v) agar plates containing one-half-strength B5 medium containing 0.03% (w/v) cefotaxime and 1% (w/v) Suc. Plants were screened for transformed roots using nucleus-localized R.GECO1.2 fluorescence. Shoots with transformed roots were grown in Agroperlite (Maasmond-Westland) supplemented with modified one-half-strength Hoagland medium (Hoagland and Arnon, 1950) containing 0.56 mm NH_4_NO_3_ and inoculated with *M. loti* R7A.pHC60 (expressing GFP) at OD_600_ = 0.05. Plants were grown at 21°C under a 16-h-light/8-h-dark regime. For calcium oscillation analysis, transformed plants were grown on one-half-strength Hoagland plates with 1% agar containing 0.56 mm NH_4_NO_3_ for 1 week. Plants were moved to N-free one-half-strength Hoagland medium 1 week prior to imaging.

### Calcium Oscillation Quantification

Calcium spiking experiments were performed on a Leica TCS SP8 HyD confocal microscope equipped with a water lens HC plan-Apochromat CS2 40×/1.0. Transformed root segments expressing R-GECO1.2 were selected and incubated with 500× diluted LCO extract (estimated to represent ∼10^−9^ m) in nitrate-free one-half-strength Hoagland medium (Hoagland and Arnon, 1950) on a glass slide with coverslip. Images were taken at 5-s intervals for a minimum of 20 min per sample using an excitation wavelength of 552 nm and emission spectrum of 585 to 620 nm. It is possible to monitor a large number of nuclei per root sample. However, only epidermal and especially root hairs were shown to be responsive. Therefore, total nuclei numbers vary largely between samples. Video recordings of imaged root samples were exported to ImageJ1.50i (Collins, 2007). The Geciquant ImageJ plugin was used for background subtraction and region of interest selection (Srinivasan et al., 2015). The average pixel intensities of regions of interest (individual nuclei) were measured. Average pixel values (0–255) per nucleus were plotted, and a background R-GECO1.2 fluorescence baseline of 2 × 1 min (two regions of 12 frames) was selected manually in a region of the trace where no spikes were occurring. Only nuclei with a minimum of three spikes with an amplitude of over 1.5 times background were considered as positive.

### *P. andersonii* Growth Conditions for Propagation, Transformation, Mycorrhization, and Nodulation

Sequenced *P. andersonii* WU1 trees or their direct descendants were used in all experiments (Op den Camp et al., 2011; van Velzen et al., 2018). Prior to transformation or transfer to tissue culture, *P. andersonii* trees were grown in a conditioned greenhouse at 28°C, 85% humidity, and a 16/8-h day/night regime. *P. andersonii* in vitro propagation, transformation, CRISPR-Cas9 mutagenesis, and nodulation assays were done according to van Zeijl et al. (2018). *P. andersonii* hairy root transformations were performed according to Cao et al. (2012).

### *P. andersonii* Nodulation Assay and Analysis

Rooted tissue culture plantlets for phenotyping assays were grown in crystal-clear polypropylene containers (1 L) with a gas-exchange filter (OS140BOX; Duchefa Biochemie). Pots were half-filled with Agroperlite (Maasmond-Westland) and watered with modified EKM medium (3 mm MES [C_6_H_13_NO_4_], pH 6.6, 2.08 mm MgSO_4_, 0.88 mm KH_2_PO_4_, 2.07 mm K_2_HPO_4_, 1.45 mm CaCl_2_, 0.7 mm Na_2_SO_4_, 0.375 mm NH_4_NO_3_, 15 μm iron citrate, 6.6 μm MnSO_4_, 1.5 μm ZnSO_4_, 1.6 μm CuSO_4_, 4 μm H_3_BO_3_, and 4.1 μm Na_2_MoO_4_; Becking, 1983). For nodulation assays, modified EKM medium (Becking, 1983) was inoculated with rhizobia (OD_600_ = 0.025) prior to planting the shoots. For inoculation with strain *S. fredii* NGR234.pHC60, containers were half-filled with sterilized river sand and watered with modified EKM medium containing the bacteria at OD_600_ = 0.05.

All nodules were fixed in buffer containing 4% (w/v) paraformaldehyde mixed with 3% (v/v) glutaraldehyde in 50 mm phosphate (pH 7.4). A vacuum was applied for 2 h during a total 48-h incubation. Fixed nodules were embedded in plastic (Technovit 7100; Heraeus-Kulzer) according to the manufacturer’s recommendations. Sections (5 µm) were made using an RJ2035 microtome (Leica Microsystems). Sections were stained using 0.05% (w/v) Toluidine Blue O. Images were taken with a DM5500B microscope equipped with a DFC425c camera (Leica Microsystems).

### *P. andersonii* Mycorrhization Assay

For mycorrhization experiments, pots were half-filled with sterilized river sand and watered with modified one-half-strength Hoagland medium containing 20 µm potassium phosphate. Pots were inoculated with 250 spores of *Rhizopagus irregularis* (Agronutrion-DAOM197198). In all experiments, plantlets in pots with closed lids were placed in a climate room at 28°C, 16/8 h day/night. Plants were watered with sterilized, demineralized water. Plants were harvested 6 wpi with *R. irregularis* (Agronutrion-DAOM197198). Root segments were treated with 10% (w/v) KOH and incubated at 90°C for 20 min. The root samples were then rinsed six times with water and stained with Trypan Blue at 90°C for 5 min. For each mutant, 10 plants were assessed, and from each plant, 30 root segments (each segment approximately 1 cm long) were examined, and mycorrhizal structures (hyphae, vesicles, and arbuscules) were determined using the magnified line intersect method (Trouvelot et al., 1986) using a Leica CTR6000 microscope. For staining with WGA-Alexa488 (Molecular Probes, Thermo Fisher Scientific), roots were incubated in 10% (w/v) KOH at 60°C for 3 h. Then, roots were washed three times in phosphate-buffered saline (150 mm NaCl, 10 mm Na_2_HPO_4_, and 1.8 mm KH_2_PO_4_, pH 7.4) and incubated in 0.2 μg mL^−1^ WGA-Alexa488 in phosphate-buffered saline at room temperature for 16 h. For RNA isolation, *P. andersonii* wild-type plants were grown according to the conditions described above. RNA was isolated according to protocols published previously (Op den Camp et al., 2011; van Velzen et al., 2018). Mock-inoculated plants were harvested as controls. Three independent biological replicates were taken per sample. Expression was determined using RNA sequencing. Reads were mapped using kallisto (Bray et al., 2016). Expression values and differential expression were determined using sleuth (Pimentel et al., 2017). Differentially expressed genes were identified using Benjamini-Hochberg multiple testing correction (*q* ≤ 0.05).

### Quantitative PCR Analysis of PanNFPi cDNA Samples

PanNFPi cDNA samples were generated previously (Op den Camp et al., 2011). Quantitative PCR was performed in 10-μL reactions using 2× iQ SYBR Green Supermix (Bio-Rad). PCR was executed on a CFX Connect optical cycler according to the manufacturer’s protocol (Bio-Rad). Three technical replicates per cDNA sample were used. Data analysis and statistical analysis of biological replicates were performed using CFX Manager 3.0 software (Bio-Rad). Gene expression was normalized against reference genes *PanACTIN* and *PanEF1α*. Primers can be found in Supplemental Table S4.

### ROS Assay

*P. andersonii* plantlets were grown on rooting medium (van Zeijl et al., 2018) for 4 weeks at 28°C before the treatment. Roots, submerged in water, were cut into approximately 1-cm pieces. Each well of a black 96-well flat-bottom polystyrene plate (Nunc) was filled with 10 root pieces. Ten replicates per line were analyzed. After filling the wells, the plate was kept 5 h in 28°C. After incubation, the water was replaced with 100 µL of assay solution containing 0.5 µm L-012 (FUJIFILM Wako Chemicals), 10 µg mL^−1^ horseradish peroxidase (Sigma), and respective elicitors (CO7; ELICITYL) or LCOs extracted from *M. loti* or *R. tropici* at the described concentrations. As a mock treatment, 100 µL of water was added. The light emission was immediately measured at 30-s intervals for 30 min using a Clariostar multi-well plate reader. All data are averages of at least three independent biological replicates.

### Protein Extraction from *P. andersonii* and Western Blotting

*P. andersonii* plantlets were grown on rooting medium (van Zeijl et al., 2018) for 4 weeks at 28°C before the treatment. About 200 mg of roots was cut while submerged in water and collected in a PCR tube. Root segments were incubated for 5 h at 28°C before treatment. Root pieces were treated with water containing 100 μm CO7 (ELICITYL) for 10 min. After incubation, roots were immediately frozen in liquid nitrogen. Samples were homogenized using metal beads. Total root protein was extracted in a buffer containing 50 mm Tris-HCl (pH 7.5), 150 mm KCl, 1 mm EDTA (pH 7.5), 0.1% (w/v) Triton X-100, 1 mm DTT, complete protease inhibitors (Roche), and phosstop (Roche). Amounts of extracted protein were measured with Qubit (Thermo Fisher Scientific), and equal amounts of protein (∼20 μg) were electrophoresed on Mini-PROTEAN TGX stain-free gels (Bio-Rad). A Trans-Blot Turbo Transfer system was used for blotting. To visualize phosphorylated MPK3/MPK6, the antibody for anti-phospho-p44/42 MAPK was used (no. 4370; Cell Signaling Technology). Anti-rabbit antibody (no. 7054; Cell Signaling Technology) was used as a secondary antibody. Equal loading was confirmed by Coomassie Brilliant Blue staining.

### Quantification and Statistical Analysis

Nodule number was quantified as mean nodule number ± se for all experiments. Replicate number is denoted in figures or figure legends. Additionally, all individual data points were plotted for graphical visualization of variation. Graphs and statistical analysis were performed using R studio 1.1.456 for nodulation experiments. Statistical tests on nodule numbers was done using one-way ANOVA and Tukey’s posthoc test for multiple comparisons. Statistical significance was defined as *P* < 0.05. Levene’s test for homogeneity of variance was used prior to running one-way ANOVA. In cases where the normality assumption was violated, alternative tests such as the Mann-Whitney-Wilcoxon test were used as denoted in the figure legends. For the mycorrhization experiment, a standard linear model was used to estimate the difference, and the corresponding lsd, of the knockout mutants with the wild-type control. The lsd with respect to the control was Bonferroni adjusted to correct for multiple testing.

### Accession Numbers

Sequence data from this article can be found in the GenBank/EMBL data libraries under the accession numbers mentioned in Supplemental Table S1.

### Supplemental Data

The following supplemental materials are available.

**Supplemental Figure S1.** Phylogeny reconstruction of orthogroups representing LysM-type receptors.
**Supplemental Figure S2.** Duplication of the *LYK3* first exon is conserved among *Parasponia* spp. and *Trema* subspecies.
**Supplemental Figure S3.** Complementation of a *L. japonicus* *Ljnfr1;Ljnfr5* mutant for LCO-induced calcium oscillation.
**Supplemental Figure S4.** Nodulation is affected in *P. andersonii Pannfp1*, *Pannfp2*, and *Panlyk3* CRISPR-Cas9 mutants.
**Supplemental Figure S5.** Complementation of the *P. andersonii Panlyk1;Panlyk3* double mutant.
**Supplemental Figure S6.** CO7 triggered ROS production and MPK phosphorylation in *P. andersonii* mutant lines.
**Supplemental Figure S7.** Expression of *P. andersonii* LysM-type receptors during mycorrhization.
**Supplemental Figure S8.** *P. andersonii* LysM-type receptor mutants can establish AM symbiosis.
**Supplemental Figure S9.** Conserved indels in NFP-II-type receptor proteins.
**Supplemental Figure S10.** The PanNFPi RNAi construct has off-target activity on *PanNFP2* and other LysM-type receptor kinases.
**Supplemental Table S1.** *P. andersonii* LysM-type receptors.
**Supplemental Table S2.** Transcomplementation of *L. japonicus* *Ljnfr1;Ljnfr5* for nodulation.
**Supplemental Table S3.** Constructs generated in this study.
**Supplemental Table S4.** Primers used in this study.
**Supplemental Data Set S1.** Sequence alignment of LYK-I-type receptors in fasta format.
**Supplemental Data Set S2.** Sequence alignment of LYR-Ia-type receptors in fasta format.
**Supplemental Data Set S3.** Genotyping and off-target analysis of *P. andersonii* CRISPR-Cas9 mutants generated in this study.
**Supplemental Movie S1.** Calcium spiking in root hairs of the *L. japonicus* *Ljnfr1-1;Ljnfr5-2* double mutant complemented with *LjNFR1;LjNFR5*.
**Supplemental Movie S2.** Calcium spiking in root hairs of the *L. japonicus* *Ljnfr1-1;Ljnfr5-2* double mutant transcomplemented with *PanLYK3.1;LjNFR5*.

## DIVE Curated Terms

The following phenotypic, genotypic, and functional terms are of significance to the work described in this paper:

calcium CHEBI: CHEBI:29320
